# Predictive Modeling of Secondary Pulmonary Hypertension in Left Ventricular Diastolic Dysfunction

**DOI:** 10.3389/fphys.2021.666915

**Published:** 2021-07-01

**Authors:** Karlyn K. Harrod, Jeffrey L. Rogers, Jeffrey A. Feinstein, Alison L. Marsden, Daniele E. Schiavazzi

**Affiliations:** ^1^Department of Applied and Computational Mathematics and Statistics, University of Notre Dame, Notre Dame, IN, United States; ^2^Department of Digital Health, T.J. Watson Research Center, International Business Machines Corporation, Yorktown Heights, NY, United States; ^3^Department of Pediatrics and Bioengineering, Stanford University, Stanford, CA, United States; ^4^Department of Pediatrics, Bioengineering and Institute for Computational and Mathematical Engineering, Stanford University, Stanford, CA, United States

**Keywords:** computational physiology, data assimilation, predictive models for pulmonary pressures, model-based disease detection, lumped parameter hemodynamic modeling, adaptive Markov chain Monte Carlo

## Abstract

Diastolic dysfunction is a common pathology occurring in about one third of patients affected by heart failure. This condition may not be associated with a marked decrease in cardiac output or systemic pressure and therefore is more difficult to diagnose than its systolic counterpart. Compromised relaxation or increased stiffness of the left ventricle induces an increase in the upstream pulmonary pressures, and is classified as secondary or group II pulmonary hypertension (2018 Nice classification). This may result in an increase in the right ventricular afterload leading to right ventricular failure. Elevated pulmonary pressures are therefore an important clinical indicator of diastolic heart failure (sometimes referred to as *heart failure with preserved ejection fraction*, HFpEF), showing significant correlation with associated mortality. However, accurate measurements of this quantity are typically obtained through invasive catheterization and after the onset of symptoms. In this study, we use the hemodynamic consistency of a differential-algebraic circulation model to predict pulmonary pressures in adult patients from other, possibly non-invasive, clinical data. We investigate several aspects of the problem, including the ability of model outputs to represent a sufficiently wide pathologic spectrum, the identifiability of the model's parameters, and the accuracy of the predicted pulmonary pressures. We also find that a classifier using the assimilated model parameters as features is free from the problem of missing data and is able to detect pulmonary hypertension with sufficiently high accuracy. For a cohort of 82 patients suffering from various degrees of heart failure severity, we show that systolic, diastolic, and wedge pulmonary pressures can be estimated on average within 8, 6, and 6 mmHg, respectively. We also show that, in general, increased data availability leads to improved predictions.

## 1. Introduction

Diastolic heart failure (sometimes referred to as *heart failure with preserved ejection fraction* or HFpEF, see Table 1) is a serious, often fatal, cardiovascular pathology. Recent reviews (Obokata et al., 2020) report that this pathology is often associated with several comorbidities, making the selection of homogeneous treatment groups difficult. It is, however, commonly characterized by elevated left ventricular filling pressures and normal systemic circulatory indicators such as left ventricular ejection fraction, cardiac output, and mean arterial pressure (Bonow and Udelson, 1992). In 2013, heart failure was mentioned in one of every nine death certificates in the United States, and was the underlying condition in roughly 20% of these cases. The number of deaths attributable to heart failure was approximately as high in 1995 as it was in 2013, with hospital discharges remaining stable from 2000 to 2010 (Mozaffarian et al., 2016). It is also estimated that about one-third of the patients with congestive heart failure (CHF) have a normal left ventricular ejection fraction (LVEF, see e.g., Zile and Brutsaert, 2002).

**Table 1:** List of acronyms.

**Acronym**	**Description**
CHF	Congestive heart failure
CVP	Central venous pressure
CO	Cardiac output
DBP	Diastolic blood pressure
dPAP	Diastolic pulmonary arterial pressure
EHR	Electronic health record
FIM	Fisher information matrix
HFpEF	Heart failure with preserved ejection fraction
HR	Heart rate
KL	Kullback-Leibler
LPN	Lumper parameter network
LVEDV	Left ventricular end diastolic volume
LVEF	Left ventricular ejection fraction
OOP	Object oriented programming
PAP	Pulmonary artery pressure
PCW	Pulmonary capillary wedge pressure
PH	Pulmonary hypertension
PVR	Pulmonary vascular resistence
NM	Nelder-Mead
MAP	Maximum *a posteriori*
MCMC	Markov chain Monte Carlo
mPAP	Mean pulmonary arterial pressure
RAP	Mean right atrial pressure
REDCap	Research electronic data capture
RC	Resistance-capacitance
RCR	Resistance-capacitance-resistance
RLC	Resistance-inductance-capacitance
RVEDP	Right ventricular end diastolic pressure
RVEF	Right ventricular ejection fraction
SBP	Systolic blood pressure
sPAP	Systolic pulmonary arterial pressure
SVR	Systemic vascular resistance

It has been observed that pulmonary hypertension (PH) is highly prevalent and often severe in HFpEF and that both pulmonary venous and arterial hypertension contribute to the severity of HFpEF with a marked correlation between systolic pulmonary arterial pressure (sPAP) and mortality (Lam et al., 2009; Obokata et al., 2020). A recent paper reviewing the current understanding of etiology and treatment of HFpEF, reports that multiple non-diastolic abnormalities contribute to the syndrome of HFpEF, including LV systolic dysfunction, pulmonary hypertension, RV and LA dysfunction, vascular stiffening, ventricular interdependence, and coronary microcirculation dysfunction (Obokata et al., 2020). Additionally, homogeneous treatment of HFpEF is made difficult by the multitude of phenotypes induced by obesity, ischemia, and cardiometabolic abnormalities. This is consistent with the findings in Shah et al. (2015) where a machine learning-based approach is used to cluster patients with HFpEF in three phenogroups with common characteristics, providing a way to go beyond a homogeneous treatment of HFpEF that has so far produced unsatisfactory results. Even though these studies highlight the current challenges in the treatment of HFpEF, they both seem to agree on the strong association between PH and HFpEF. In this regard, Obokata et al. (2020) mentions how “PH is extremely common in HFpEF, seen in roughly 80% of patients, and mortality is increased in this cohort,” whereas in Shah et al. (2015), elevated PH was one of the main criteria for patient recruitment.

While non-invasive echocardiography and machine learning may be useful for phenotyping classification and treatment selection (Obokata et al., 2020), early diagnosis of HFpEF relies on invasive pressure acquisition through right-heart catheterization, often performed following the manifestation of symptoms (Fisher et al., 2009; Galiè et al., 2009). Therefore, it is evident that methods enabling accurate indirect estimation of pulmonary pressures using minimally invasive clinical data would be extremely beneficial for early diagnosis of HFpEF in a way that could trigger lifestyle changes that will, in turn, prevent other co-morbidities from developing. Thus, in this study, we investigate how the physics-based consistency of a lumped parameter hemodynamic model containing three compartments, i.e., a four-chamber heart, systemic and pulmonary circulation compartments, may be used to monitor PH in patients from non-invasive and uncertain clinical measurements.

The development of computer models to study hemodynamics in humans started in the 1960s and 1980s (Snyder and Rideout, 1969; Avanzolini et al., 1985, 1988; Ursino, 1998), with application in pediatrics developed in the 2000s for single-ventricle congenital heart disease (Pennati et al., 1997), Norwood physiology (Migliavacca et al., 2001), and systemic-to-pulmonary artery shunts (Pennati et al., 2010). Approaches for automatic parameter estimation date back to the late 1970s (Deswysen, 1977; Deswysen et al., 1980), ranging from two-stage Prony-Marquardt optimization (Clark et al., 1980) and adaptive control systems for left ventricular bypass assist devices (McInnis et al., 1985; Shimooka et al., 1991) to Kalman filters (Yu et al., 1998, 2001) and recursive least squares (Ruchti et al., 1993). An iterative, proportional gain-based identification method is presented in Revie et al. (2013) and an application to coronary artery disease is discussed in Sughimoto et al. (2013). Other studies include estimation of three-element Windkessel boundary conditions (Spilker and Taylor, 2010) and left ventricular viscoelasticity (Cappello et al., 1987; Avanzolini et al., 1992). More recently, examples of automatic parameter tuning in lumped circulatory models have included the physiology of children with congenital heart disease undergoing the first stage (i.e., Norwood) of single ventricle palliation surgery (Schiavazzi et al., 2016), construction of optimally trained patient-specific models for coronary artery disease (Tran et al., 2017) and predicting time evolution of ventricular dilation and thickening (Witzenburg and Holmes, 2018). A study using lumped parameter models in diastolic heart failure is finally discussed in Luo et al. (2011), while parameter identification for a mice model with chronic hypoxia and drug-induced pulmonary hypertension is proposed in Tewari et al. (2013).

Circuit models in hemodynamics typically contain a large number of parameters which need to be trained from clinical records collected at multiple visits, including a variable but typically sparse number of clinical measurements. This aggravates the ill-conditioning of the inverse problem, where model outputs do not change in response to perturbations along a number of unidentifiable linear combinations of parameters. In these circumstances, optimization may not be successful in identifying global optima and sequential Monte Carlo techniques (Del Moral et al., 2006) may underperform in practice, as data typically represent extremes (maxima/minima) or mean values of clinical indicators over one heart cycle.

Two technological trends make the present contribution particularly timely. On the one hand, there is increasing importance attributed to the availability of large training datasets which is at the base of the current revolution in AI and deep learning (see, e.g., LeCun et al., 2015). This includes anonymous electronic health records (EHRs) for specific sub-populations affected by a common clinical condition. On the other hand, there is an increasing availability of computational resources on the cloud, creating a perfect infrastructure for distributed computing with lightweight models. For these reasons, we envision an increased adoption of numerical models as *regularizers* to determine physics-informed predictive distributions for missing data in EHRs, going beyond currently adopted, physics-agnostic multiple imputation methods (Schafer, 1999). This study aims to be the first step in this direction and provides the following new contributions:

- We propose a systematic approach to train patient-specific circulatory models with clinical data uncertainty, and demonstrate the results obtainable on a modest cohort of 82 patients.
- Explore optimal parameter training as a possible approach to increase the feature space in order to facilitate classification of cardiovascular anomalies.

In section 2.2, we discuss the differential formulations of a compartmental circulation model for human adults, including circuit elements, a generic heart model, and the governing equations for the aortic, systemic, and pulmonary compartments. This is followed by an analysis of two datasets in section 2.3, the first used for validation, while the second contains EHRs for 82 patients. Our numerical investigation is articulated through answers to the questions formulated in section 2.4, using the numerical algorithms and tools briefly introduced in sections 2.5 and 2.6. Section 3 highlights the results of both the model analysis and the predictive results of the model. This section addresses the physiological admissibility of the selected model, the ability of the model to capture dysfunction mechanisms, and the sensitivity and identifiability of input parameters. On the predictive side, this section addresses the predictive performance of pulmonary pressures, non-pulmonary target ranking, and viability of model-based PH classifiers. Conclusions are discussed in section 4. Finally, for convenience, a list of acronyms is provided in Table 1.

## 2. Materials and Methods

### 2.1. Compliance With Ethical Standards

This study was classified as research not involving human subjects and was approved on June 13th, 2019, by the Office of Research Compliance and Institutional Review Board at the University of Notre Dame under IRB#19-05-5371. This work utilizes data resulting from external studies that involved human participants. The medical procedures that occurred in these studies were performed in accordance with both the ethical standards of the institutional and national research committee and with the 1964 Helsinki declaration and its later amendments. As this study is a retrospective study, formal consent from the involved human participants was not required.

### 2.2. Modeling Approach

**Figure 1:**
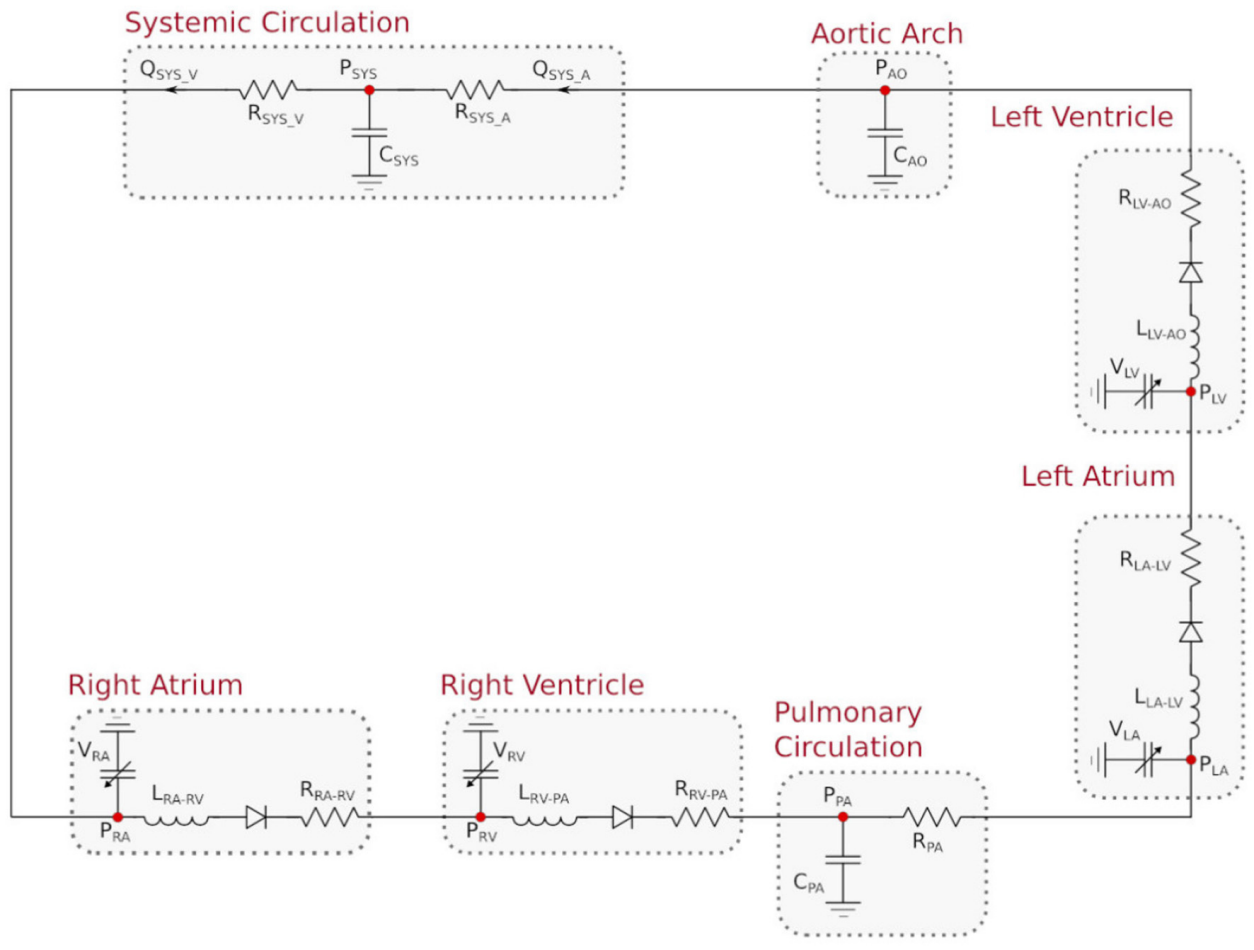
Lumped parameter hemodynamic model with RC pulmonary circuit.

Blood circulation in adults can be simulated through a *lumped parameter model* (sometimes also referred to as *zero-dimensional* or *0D* model). The foundation beneath a lumped parameter model lies in the equations that govern the voltage and current in an electrical circuit. The equations that underlie the voltage and current of an electrical circuit stem from the principles of the conservation of energy. Utilizing the hydrodynamic analogy (see, e.g., Rideout and Dick, 1967), these equations can also be used to govern the pressure and flow of blood where voltage corresponds to pressure and current corresponds to flow. In this study, we consider patient-specific 0D representations containing seven compartments: the four chambers in a bi-ventricular heart, a compliant aortic arch, and the pulmonary and systemic circulations. The pulmonary compartment is represented through an RC circuit, as shown in Figure 1.

#### 2.2.1. Generic Heart Model Compartment

The four heart chambers are represented by a series arrangement of a pressure-volume generator, an inductor, an ideal unidirectional valve, and a resistor, following prior work. The atrial and ventricular pressure-volume generators are formulated through a combination of an activation function and active/passive pressure curves (Avanzolini et al., 1985; Migliavacca et al., 2001),

(1) $$\begin{array}{l} P_{i,act}=E_{i,act}\left(V_{i}-V_{i,0}\right),\\ P_{i,pas}=K_{i,pas,1}\left[e^{K_{i,pas,2}\left(V_{i}-V_{i,0}\right)}-1\right],P_{i}=P_{i,pas}+A_{i}P_{i,act}, \end{array}$$

where the index *i* refers to either the right-left or atrial-ventricular chamber, *i* ∈ {*ra, rv, la, lv*}. The active pressure curve is assumed linear and characterized by an *active* elastance *E*_*i,act*_ and an *unstressed chamber volume* *V*_*i*,0_. Additionally, passive volumes and pressures are related through an exponential relation, characterized by two elastance coefficients *K*_*i,pas*,1_ and *K*_*i,pas*,2_ (Avanzolini et al., 1985; Migliavacca et al., 2001). This is the simplest formulation compatible with the finite strains experienced by the ventricle during filling (see, e.g., Mirsky, 1976). The pressure-volume generator is completed by an atrial and ventricular *activation functions* *A*_*a,i*_, *A*_*v,i*_ of the form

(2) $$\begin{array}{l} A_{a,i}=\{\begin{array}{ll} \frac{1}{2}\left[1-cos\left(2\pi \frac{t_{ma}}{t_{sa}}\right)\right], & \text{if }t_{ma}<t_{sa}\\ 0, & \text{otherwise}, \end{array},\\ A_{v,i}=\{\begin{array}{ll} \frac{1}{2}\left[1-cos\left(2\pi \frac{t_{mv}}{t_{sv}}\right)\right], & \text{if }t_{mv}<t_{sv}\\ 0, & \text{otherwise}, \end{array} \end{array}$$

where *t*_*sa*_ = *t*_*c*_ · *t*_*sas*_ and *t*_*sv*_ = *t*_*c*_ · *t*_*svs*_ are the atrial and ventricular relative activation times, respectively, and *t*_*c*_ = 60.0/*HR* is the heart cycle duration in seconds. Additionally, *t*_*mv*_ = *t* − *t*/*t*_*c*_*t*_*c*_ is the time from the beginning of systole (start of the cardiac cycle), *t*_*pw*_ = *t*_*c*_/*t*_*pws*_ is the atrial relative activation time delay and *t*_*ma*_ = (*t* + *t*_*sa*_ − *t*_*pw*_) − (*t* + *t*_*sa*_ − *t*_*pw*_)/*t*_*c*_*t*_*c*_. The model inputs are *t*_*sas*_, *t*_*svs*_ and *t*_*pws*_. The chamber volume is determined through the equations

(3) $$\begin{array}{l} \begin{array}{c} \frac{dV_{ra}}{dt}=Q_{sys,v}-Q_{ra,rv}\cdot \phi_{T},\frac{dV_{rv}}{dt}=Q_{ra,rv}\cdot \phi_{T}-Q_{rv,pa}\cdot \phi_{P},\\ \frac{dV_{la}}{dt}=Q_{pul}-Q_{la,lv}\cdot \phi_{M},\frac{dV_{lv}}{dt}=Q_{la,lv}\cdot \phi_{M}-Q_{lv,ao}\cdot \phi_{A}, \end{array} \end{array}$$

where ϕ_*i*_, *i* ∈ {*T, P, M, A*} are valve activation functions for the tricuspid (T), pulmonary (P), mitral (M), and aortic (A) valve, respectively. These are equal to one for a negative pressure gradient through the valve and zero otherwise. Moreover, valves are modeled as *perfect*, without accounting for possible leakage or regurgitation. For models including valve prolapse and consequent regurgitation the reader is referred to, e.g., Pant et al. (2016). An inductance element located downstream with respect to the pressure-volume generator simulates the inertia of the blood in the chamber, according to the differential equation

(4) $$\{\begin{array}{ll} dQ_{i}/dt=(P_{i}-P_{dn}-R_{i}Q_{i})/L_{i}, & \text{if }P_{i}\geq P_{dn}\\ 0, & \text{otherwise}, \end{array}$$

where *Q*_*i*_ is the volumetric blood flow going through the *i*-th chamber, *P*_*i*_ and *P*_*dn*_ the pressure in the *i*-th and downstream chamber, respectively, *R*_*i*_ the viscous resistance located between chamber *i* and chamber *dn*, and *L*_*i*_ an inductance parameter.

Additionally, the selected model is fully capable of representing the physiologic consequences of a stenotic valve, as an increase in the resistance of the associated compartment would accentuate the pressure drop across the valve and lead to an increase of the upstream pressure. Finally, we remark that systolic and diastolic functions are separately represented in this model, using three parameters for each chamber, i.e., *E*_*i,act*_, *K*_*i,pas*,1_, and *K*_*i,pas*,2_. Identification of these parameters from clinical health records would therefore be informative of systolic and diastolic chamber function.

Note that the RL parameters of each cardiac chamber remain fixed in this study (see Supplementary Table 1). In other words, we assume that valvular stenosis has been excluded as a possible cause of pulmonary hypertension, for example, exclusion through a non-invasive echocardiographic assessment.

#### 2.2.2. Aortic and Systemic Compartment

An aortic compartment consisting of an isolated capacitor is positioned downstream of the left ventricular outflow, modeled through an equation of the form

(5) $$\begin{array}{l} \frac{dP_{ao}}{dt}=\frac{Q_{up}-Q_{dn}}{C_{ao}}, \end{array}$$

where *Q*_*up*_ and *Q*_*dn*_ are the volumetric flow rate from the left ventricle and abdominal aorta, respectively, *P*_*ao*_ is the aortic pressure and *C*_*ao*_ the aortic compliance. We compare the value of *P*_*ao*_ computed by this model with the clinically acquired brachial pressure.

An RCR circuit simulates the systemic circulation, with *C*_*sys*_ used to represent the overall systemic compliance, while two resistors simulate the viscous resistance in arteries and veins *R*_*sys,a*_ and *R*_*sys,v*_, respectively. The algebraic-differential equations for the systemic compartments are therefore:

(6) $$\begin{array}{l} Q_{sys,a}=\frac{P_{ao}-P_{sys}}{R_{sys,a}},\;Q_{sys,v}=\frac{P_{sys}-P_{ra}}{R_{sys,v}},\\ \;\frac{dP_{sys}}{dt}=\frac{Q_{sys,a}-Q_{sys,v}}{C_{sys}}. \end{array}$$

#### 2.2.3. Pulmonary Compartment

The pulmonary circulation is represented through a RC circuit with equations

(7) $$\begin{array}{l} Q_{pa}=\frac{P_{pa}-P_{la}}{R_{pa}},\;\frac{dP_{pa}}{dt}=\frac{Q_{rv,pa}\cdot \phi_{P}-Q_{pa}}{C_{pa}}, \end{array}$$

where the pulmonary, left atrial and right ventricular pressures are denoted by *P*_*pa*_, *P*_*la*_, and *P*_*rv*_, and pulmonary capacitance and resistance are *C*_*pa*_ and *R*_*pa*_, respectively. The pulmonary flow rate is denoted as *Q*_*pa*_, while *Q*_*rv,pa*_ indicates the flow across the pulmonary valve, having activation equal to,

(8) $$\phi_{P}=\{\begin{array}{ll} 0 & \text{if}P_{pa}\geq P_{rv}\\ 1 & \text{otherwise} \end{array}$$

#### 2.2.4. Initial Conditions

Initial conditions are specified for all state variables in the system of ODE. These include ventricular and atrial chamber volumes (*V*_*rv,ini*_, *V*_*lv,ini*_, *V*_*ra,ini*_, *V*_*la,ini*_). Additionally, the model contains one state variable for every capacitor and inductor. Inductors are located at each valve, so initial conditions must be specified within the model for the initial flow across the tricuspid (*Q*_*ra,rv*_), pulmonary (*Q*_*rv,pa*_), mitral (*Q*_*la,lv*_), and aortic (*Q*_*lv,ao*_) valve. Finally, an initial pressure is specified for capacitors located in the aorta (*P*_*ao*_), pulmonary (*P*_*pa*_) and systemic (*P*_*sys*_) circulation.

### 2.3. Available Data Sets

Two data sets are used throughout this study. Synthetic patient-agnostic clinical measurements representing increasing severity of diastolic left ventricular dysfunction are used initially, while anonymized patient-specific electronic health records (EHR) for a cohort of 82 patients are utilized in the second part of the study.

#### 2.3.1. Validation Data Set

The validation data set (Table 2) contains the mean and standard deviation of thirteen different clinical targets for three different heart failure groups: healthy patients, mild heart failure patients, and patients with severe heart failure. Normal physiologic clinical targets were determined from the literature (Edwards Lifesciences Corporation, 2009), while targets associated with severe left ventricular diastolic dysfunction were assigned with the supervision of a clinician. The values for mild heart failure patients were obtained through a linear interpolation between severe dysfunction and healthy conditions. The selected targets for severe HF conditions are characterized by a normal SBP and DBP accompanied by a slight reduction in CO.

**Table 2:** Validation data set containing the mean and standard deviation of clinical targets for three levels of increasing diastolic heart failure severity.

**Qty**	**Description**	**Units**	**Severe HF**	**Moderate HF**	**Healthy**	**σ**
HR	Heart rate	bpm	80	80	80	3
RAP	Right atrial pressure	mmHg	15	9	4	0.5
sPAP	Systolic pulmonary artery pressure	mmHg	50	35	20	1
dPAP	Diastolic pulmonary artery pressure	mmHg	25	19	12	1
PCW	Pulmonary capillary wedge pressure	mmHg	25	17	9	1
SBP	Systolic blood pressure	mmHg	120	120	120	1.5
DBP	Diastolic blood pressure	mmHg	80	80	80	1.5
SVR	Systemic vascular resistance	dynes·s·cm^−5^	1800	1575	1350	50
CO	Cardiac output	L/min	3.5	4.375	5.25	0.2
sRV	Systolic right ventricular pressure	mmHg	50	35	20	1
RVEDP	Right ventricular end diastolic pressure	mmHg	15	9	4	1
sLV	Systolic left ventricular pressure	mmHg	120	120	120	1.5
LVEDP	Left ventricular end diastolic pressure	mmHg	25	16	6	2

#### 2.3.2. EHR Data Set

**Figure 2:**
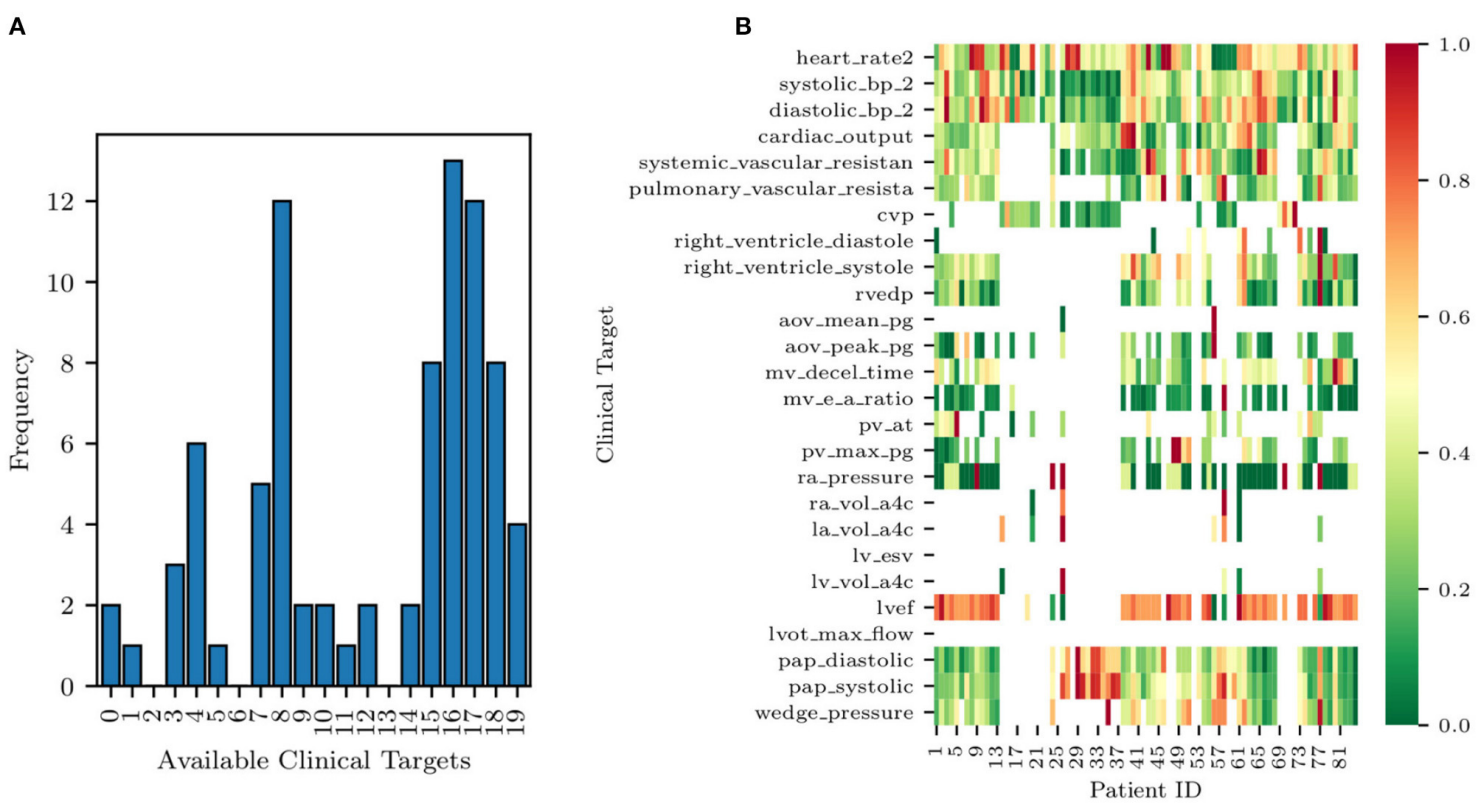
Histogram of data availability among the 82 patients **(A)** and missing data pattern **(B)**. Note how each row of the heat map is normalized to a zero to one range, highlighting the relative magnitude of the clinical target.

Completely anonymized patient-specific clinical measurements for 82 adult patients were provided in the context of a research project funded by Google through its ATAP initiative, focusing on *Modeling Non-invasive Measurements of Cardiovascular Dynamics*. There are 26 clinical data targets, which are listed below in Table 3. Missing data were present with the pattern highlighted in Figure 2, with patients having zero to nineteen of the clinical targets. Two of the 84 patients in the dataset did not have any of the relevant clinical targets and were therefore excluded from the study (see the two patients with zero available targets in Figure 2A). The remaining 82 patients had between one and nineteen clinical targets. All patients but one had measurements for three clinical targets: heart rate, diastolic blood pressure, and systolic blood pressure. No single patient had all 26 measurements. Finally, the standard deviations for each target are also shown in Table 3. Their value was determined through a preliminary literature review (see, e.g., Gordon et al., 1983; Maceira et al., 2006; Yared et al., 2011).

**Table 3:** Patient-specific EHR data set.

**N**.	**REDCap token**	**Description**	**Units**	**Measurement type**	**σ**	**N**.
1	heart_rate2	Heart rate	bpm	NI	3.0	81
2	systolic_bp_2	Systolic blood pressure	mmHg	NI	1.5	81
3	diastolic_bp_2	Diastolic blood pressure	mmHg	NI	1.5	81
4	cardiac_output	Cardiac output	L/min	Invasive TD or NI echo	0.2	65
5	systemic_vascular_resistan	Systemic vascular resistance	dynes·s·cm^−5^	from RAP, SBP, and CO	50.0	64
6	pulmonary_vascular_resista	Pulmonary vascular resistance	dynes·s·cm^−5^	from PAP, PCW, and CO	5.0	50
7	cvp	Central venous pressure	mmHg	NI (CVP≈RAP) or JVP/IVC CI	0.5	30
8	right_ventricle_diastole	Right ventricle diastolic pressure	mmHg	Invasive catheter	1.0	11
9	right_ventricle_systole	Right ventricle systolic pressure	mmHg	NI echo and invasive catheter	1.0	46
10	rvedp	Right ventricle EDP	mmHg	NI same as RAP with no TS	1.0	46
11	aov_mean_pg	Average PG across aortic valve	mmHg	NI echo	0.5	2
12	aov_peak_pg	Peak PG across aortic valve	mmHg	NI echo	0.5	37
13	mv_decel_time	Mitral valve deceleration time	ms	NI echo	6.0	41
14	mv_e_a_ratio	Mitral valve E/A ratio	–	NI echo	0.2	39
15	pv_at	Pulmonary valve acceleration time	ms	NI echo	6.0	18
16	pv_max_pg	Peak PG across pulmonary valve	mmHg	NI echo	0.5	31
17	ra_pressure	Mean right atrial pressure	mmHg	NI (CVP≈RAP) or JVP/IVC CI	0.5	50
18	ra_vol_a4c	Right atrial volume	mL	NI echo	3.0	4
19	la_vol_a4c	Left atrial volume	mL	NI echo	3.0	7
20	lv_esv	Left ventricular end systolic volume	mL	NI echo	10.0	1
21	lv_vol_a4c	Left ventricular volume	mL	NI echo	20.0	5
22	lvef	Left ventricular ejection fraction	–	NI echo	2.0	53
23	lvot_max_flow	Peak flow velocity across LVOT	cm/s	NI echo	–	0
24	pap_diastolic	Diastolic PAP	mmHg	Invasive catheter	1.0	65
25	pap_systolic	Systolic PAP	mmHg	Invasive catheter	1.0	65
26	wedge_pressure	Pulmonary wedge pressure	mmHg	Invasive catheter	1.0	50

*NI, non-invasive; echo, Doppler echocardiography; PAP, pulmonary arterial pressure; JVP, jugular vein pressure; IVC CI, Inferior vena cava collapsibility index; RAP, right atrial pressure; TD, thermo dilution; PG, pressure gradient; EDP, end diastolic pressure; LVOT, left ventricular out flow tract; TS, tricuspid stenosis*.

A histogram of clinical data occurrences is illustrated in Figure 2A and displays three main modes. Most patients have either four, eight, or 17 available clinical measurements, likely due to the data aggregation produced by screening protocols. Additionally, a heat map of the EHRs is illustrated in Figure 2B. Each row of the heat map was normalized to a zero to one range to highlight the relative magnitude of the clinical target.

Note how the size of the selected cohort (84 patients) is modest but sufficient to investigate the effectiveness of Bayesian inference in the context of a simple physiologic model. Acquisition of larger data sets is possible but made non-trivial by the need to automatically extract large volumes of clinical targets from text reports written following echocardiographic and catheter lab examinations. In such cases, using natural language processing tools is key to make larger EHR data sets available for research.

Finally, this data set focuses on cases of secondary PH, where a reversible increase of PVR follows an increase in left ventricular filling pressures. Therefore, this study does not consider primary PH and does not make any claim of differentiating primary from secondary PH.

### 2.4. Methodological Approach

This study is articulated through a number of logically consequential questions driving our numerical experiments. These questions are:

1. **Physiological admissibility of 0D representations under normal and heart failure conditions**—*Are the model outputs able to reproduce sets of clinical targets ranging from healthy to pathological conditions?* In other words, is the identification problem *well-posed*, in the sense that model outputs are able to represent a wide spectrum of conditions from health to disease? We answer this question in section 3.1.
2. **Ability to model distinct diastolic/systolic dysfunction mechanisms**—*Is the selected model formulation able to separately represent the systolic and diastolic functions of the heart muscle?* And does the alteration of these properties produce expected modifications in the physiology represented through model outputs? We answer this question in section 3.2.
3. **Parameter sensitivity and identifiability**—Once a set of quantities whose prediction is of interest (e.g., pulmonary arterial pressure) has been identified, *do they show non-negligible sensitivity with respect to changes in the parameters associated with physiologically relevant mechanisms affecting these quantities*? Moreover, are these parameters *identifiable* so that it is possible to uniquely estimate their distribution from the available clinical data? Is this estimate robust (i.e., characterized by a limited uncertainty)? We answer this question in section 3.3.
4. **Predictive ability of optimally trained models**—*Are models trained from clinical data other than the pulmonary pressures able to predict such pressures, and what is their accuracy*? We answer this question in section 3.4.
5. **Relative importance of non-pulmonary clinical targets**—*Which minimal set of clinical targets should be collected to guarantee accurate model predictions for systolic, diastolic pulmonary pressure and pulmonary venous wedge pressure*? In other words, we would like to *rank* the non-pulmonary targets, starting with those producing maximally accurate predictions on the pulmonary arterial pressure. This allows for identification of a minimal set of maximally informative clinical quantities for predicting specific model outputs. We answer this question in section 3.5.
6. **Detecting pulmonary arterial hypertension from assimilated circulation models**—Group II pulmonary arterial hypertension is typically detected by a mean pulmonary pressure higher than 25 mmHg or a systolic pulmonary pressure higher than 35 mmHg (Simonneau et al., 2013). *Instead of direct characterization based on clinical data, would it be possible to detect pulmonary arterial hypertension by classification from the parameters of a model trained with non-invasive measurements*? Once assimilated, a model can be used to generate a large number of features, leading to a higher dimensional space with possibly improved separability (Cover, 1965). We answer this question in section 3.6.

### 2.5. Inference

Consider a set of *m* measured clinical targets represented through the random vector **d** ∈ ℝ^*m*^ with each component *d*_*i*_ ~ ρ_*i*_(*d*_*i*_), *i* = 1, …, *m* and joint density **d** ~ ρ(**d**) = ρ_1_(*d*_1_)ρ_2_(*d*_2_)⋯ρ_*m*_(*d*_*m*_). In other words, each quantity *d*_*i*_, *i* = 1, …, *m* measured in the clinic has marginal density ρ_*i*_(*d*_*i*_), *i* = 1, …, *m*, and we assume all these measurements to be independent, i.e., their joint probability factors. We design a physiological 0D circuit model with *n* parameters **y** ∈ ℝ^*n*^, so its outputs match the observed targets or, in other words, we introduce a statistical model of the form

(9) $$\begin{array}{l} d_{i}=G_{i}\left(\text{y}\right)+\epsilon_{i}=o_{i}+\epsilon_{i},i=1,\ldots ,m, \end{array}$$

and assume each noise component $\epsilon_{i}\sim N\left(0,\sigma_{i}^{2}\right)$ to follow a zero-mean Gaussian distribution. Note that the *i*-th realization from the parameter vector **y** is denoted by **y**^(*i*)^ and that the *i*-th model output is denoted by *o*_*i*_ = *G*_*i*_(**y**), while the vector **o** ∈ ℝ^*m*^ contains the complete set of model outputs.

Each model is *trained* using two different approaches, i.e., by determining a maximum *a posteriori* estimate of the parameters **y** using repeated Nelder-Mead optimization (Nelder and Mead, 1965), and by solving an inverse problem through adaptive Markov chain Monte Carlo sampling, specifically, through the differential evolution adaptive Metropolis algorithm (Vrugt et al., 2009; Vrugt, 2016) and assessing convergence through the Gelman-Rubin diagnostic (Gelman and Rubin, 1992). In both cases, the posterior distribution *P*(**y**|**d**) is obtained by combining a uniform prior *P*(**y**) (see the admissible parameter ranges in Supplementary Table 1) with a Gaussian likelihood,

(10) $$\begin{array}{l} P\left(\text{y}|\text{d}\right)\propto P\left(\text{d}|\text{y}\right)\cdot P\left(\text{y}\right),\\ P\left(\text{d}|\text{y}\right)=\frac{1}{\sqrt{{\left(2\pi \right)}^{m}\prod_{i=1}^{m}\sigma_{i}^{2}}}\exp\left(-\frac{1}{2}\overset{m}{\underset{i=1}{\sum }}\frac{{\left[d_{i}-G_{i}\left(\text{y}\right)\right]}^{2}}{\sigma_{i}^{2}}\right). \end{array}$$

### 2.6. Computational Tools

The TULIP (Tools for Uncertainty quantification, Lumped modeling and Identification of Parameters) software framework was developed to answer the above research questions. Tulip is a OOP C++ code designed to simplify the task of estimating parameters of lumped models for human circulation and contains abstractions for computational models, operations performed on these models (e.g., optimization, Bayesian estimation, local and global sensitivity analysis, etc.) and data sources used to store the available clinical targets. For an overview of the procedures for statistical data assimilation used in this study, the interested reader is referred to Schiavazzi et al. (2016) and Akintunde et al. (2019).

## 3. Results

### 3.1. Physiological Admissibility

**Figure 3:**
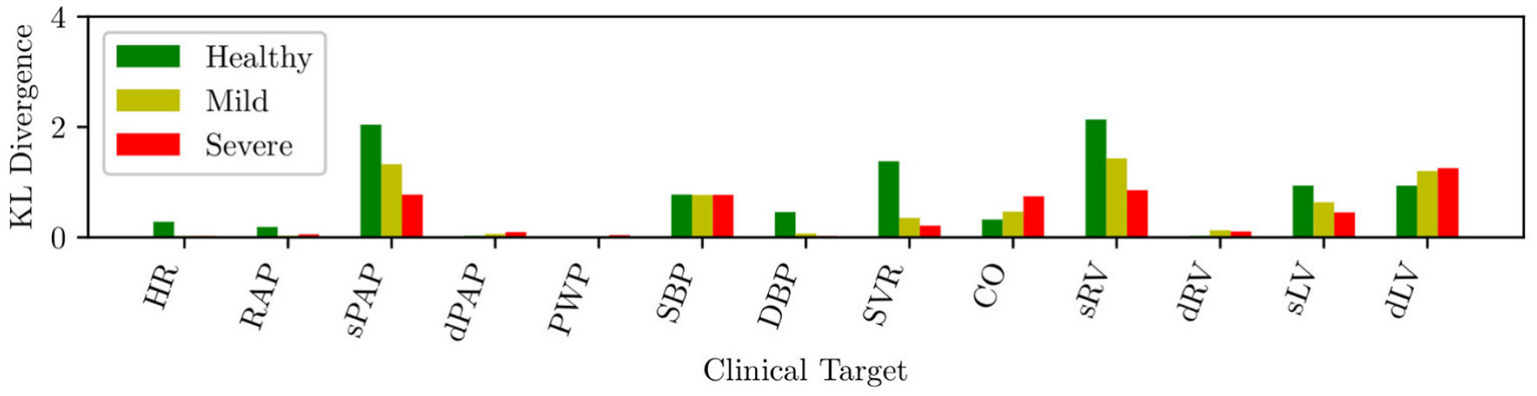
KL divergence between predicted and assumed clinical targets for varying heart failure severity.

The circulation model discussed in section 2.2 was first trained on the validation dataset in section 2.3.1. We then generated a collection of model outputs using a subset **y**^(*i*)^, *i* = 1, …, 5, 000 parameter realizations from the converged MCMC samples, and compared the resulting distributions (after post-processing with Gaussian kernel density estimation) with the distributions assumed for the targets **d**. Specifically, we assumed that each clinical target follows a normal distribution with the mean and standard deviations listed in Table 2. The Kullback-Leibler (KL) divergence (Kullback and Leibler, 1951) was used to determine the agreement between the model-based predictions and measurements. The KL divergence measures how much information is lost when one uses the predicted, instead of the assumed, target distribution and a small KL divergence suggests physiological admissibility. As shown in Figure 3, the KL divergence is negligible for all targets, and the relative percent difference between mean target value and MAP model outputs is also generally small (see Table 4).

**Table 4:** Percent differences between model outputs for MAP parameter estimates and clinical targets in the validation data set, for various degrees of heart failure severity.

	**Healthy**			**Mild**			**Severe**		
	**Target**	**Computed**	**Error (%)**	**Target**	**Computed**	**Error (%)**	**Target**	**Computed**	**Error (%)**
HR	80	80.8	0.94	80	80.6	0.81	80	81.3	1.63
RAP	4	4.1	1.70	9	9.0	0.21	15	15.0	0.19
sPAP	20	18.7	6.59	35	33.0	5.70	50	49.3	1.49
dPAP	12	11.9	0.84	19	18.6	2.34	25	24.4	2.22
PCW	9	9.1	1.65	17	16.4	3.80	25	25.1	0.34
SBP	120	118.3	1.43	120	116.6	2.84	120	117.4	2.15
DBP	80	79.5	0.61	80	78.4	1.96	80	78.9	1.37
SVR	1350	1397.4	3.51	1575	1594.8	1.26	1800	1819.7	1.09
CO	5.25	5.4	2.62	4.375	4.4	0.28	3.5	3.6	2.51
sRV	20	21.4	6.79	35	35.4	1.15	50	50.0	0.02
RVEDP	4	4.2	4.96	9	8.5	5.46	15	15.2	1.27
sLV	120	121.4	1.18	120	118.9	0.94	120	119.9	0.07
LVEDP	6	4.9	19.04	16	15.1	5.38	25	23.1	7.50

### 3.2. Inverse Assessment of Dysfunction Mechanisms

We now focus on the ability of our model to distinguish between diastolic and systolic dysfunction mechanisms when trained based on conditions reported in Table 2. Specifically, we aim to determine whether the selected model parameterization is able to separately represent the relaxation or contraction (i.e., diastolic or systolic) function of the heart muscle. Recall that diastolic function, and therefore left ventricular stiffness, during relaxation relate to the linear and exponential passive curve parameters *K*_*i,pas*,1_ and *K*_*i,pas*,2_ in equation (1). These two parameters directly relate to the condition that we aim to assess.

**Figure 4:**
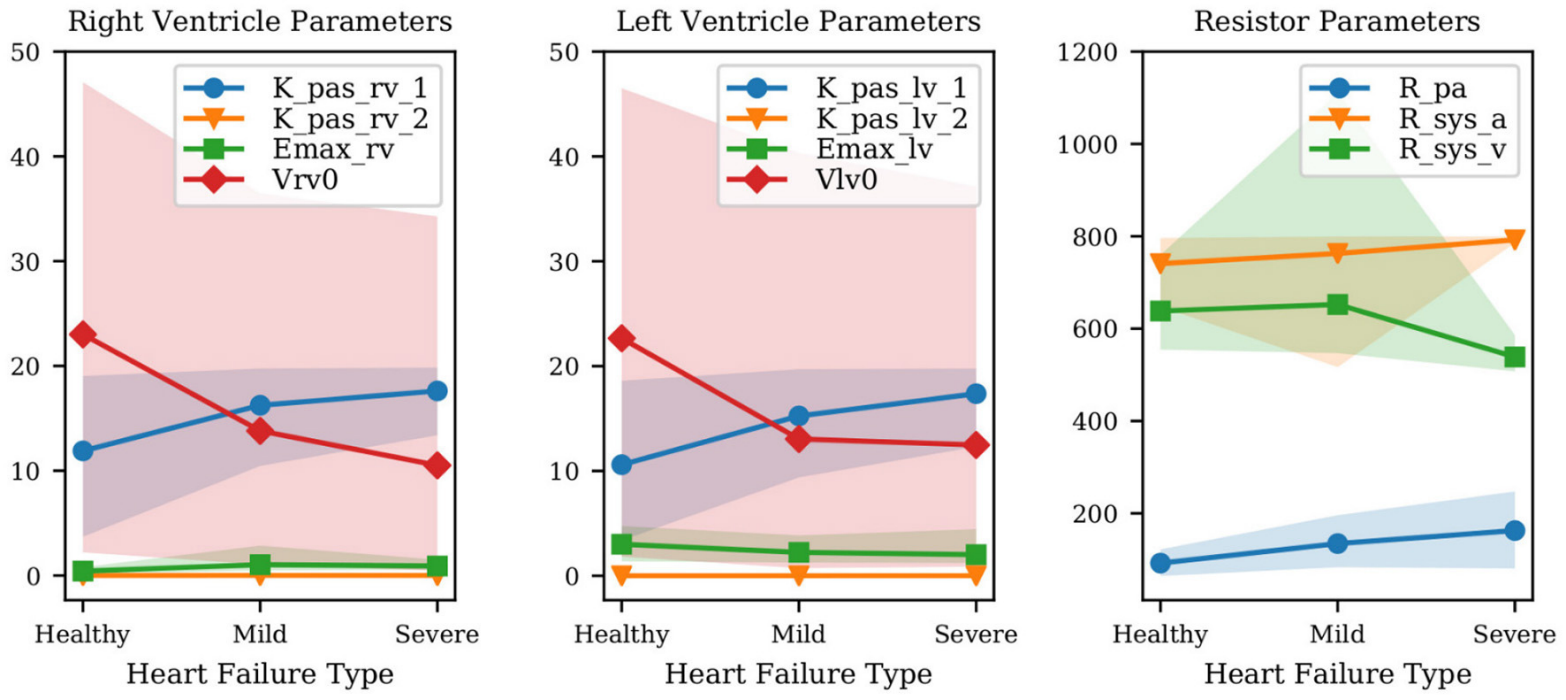
Variation of mean parameter values and associated confidence intervals under increasing heart failure severity. Right and left ventricular model parameters shown in the Figure are the ventricular passive curve slope *K*_*pas,i*,1_, exponent factor *K*_*pas,i*,2_, active curve slope *E*_*max,i*_ and unstressed ventricular volume *V*_*i*,0_ (where *i* ∈ {*rv, lv*}). Also shown: *R*_*pa*_ (pulmonary resistance), *R*_*sys,a*_ (arterial systemic resistance), *R*_*sys,v*_ (venous systemic resistance).

Figure 4 shows the mean diastolic and systolic chamber function parameters and associated 10–90% confidence intervals, grouped by anatomical relevance (i.e., left ventricle, right ventricle) and plotted for healthy patients and patients with mild and severe heart failure, respectively. Comparison between heart failure conditions allows one to assess how model parameters change due to disease progression. This change is shown in Figure 4, where many of the parameters related to pulmonary hypertension change as patients progress from healthy to mild, and mild to severe diastolic dysfunction.

The results show that the passive left ventricular curve slope *K*_*lv,pas*,1_ and the pulmonary resistance increase from healthy to mild and mild to severe PH. Additionally, changes in the left ventricular active elastance parameter *E*_*max,lv*_ remain limited, i.e., the model correctly predicts a practically unaltered systolic function. This confirms the ability of the physiological model used in this study to link PH with diastolic dysfunction and increased pulmonary resistance. Since Figure 4 combines quantities with different units, we have also looked at the left ventricular active and passive pressure-volume slope at *V*_0_. The active curve slope at *V*_0_ is equal to *E*_*max,lv*_, whose MAP estimates are 3.01, 2.23, 2.02 Barye/mL for healthy, mild, and severe PH, respectively. The passive curve slope at *V*_0_ is instead *K*_*pas,lv*,1_ * *K*_*pas,lv*,2_ (see Equation 1) equal to 0.06, 0.12, 0.15 Barye/mL. Therefore, from healthy to mild PH the active slope changes by −25.8%, while the passive slope changes by +113.1%. From mild PH to severe PH, the active slope drops by −9.4%, while the passive slope increases by +24.3%. This perspective supports our conclusion that our model consistently captures increased severity in underlying PH through more relevant variations in the diastolic rather than systolic left ventricular properties. Finally, the significant variability in the unstressed ventricular volumes is explained by the presence of multiple local peaks in the posterior distribution, i.e., reasonable PV loops compatible with the clinical targets produced by wildly different unstressed volumes.

### 3.3. Sensitivity and Identifiability of Circulation Model Parameters

Results from the above sections confirm the *well-posedness* of the selected model formulation for the full spectrum of diastolic dysfunction, from mild to severe. We now focus on determining the most relevant model parameters that significantly alter the main quantities of interest, particularly the systolic, diastolic, and pulmonary wedge pressures. In addition, we study both the structural and practical identifiability in an effort to determine unimportant parameters and their non-identifiable combinations.

#### 3.3.1. Average Local Sensitivities

Non-dimensional local sensitivities are computed for all outputs as

(11) $$\begin{array}{l} \frac{\Delta o_{i}}{\Delta y_{i}}\cdot \frac{y_{i,\text{map}}}{o_{i,\text{map}}},\text{where}\frac{\Delta y_{i}}{y_{i,\text{map}}}=0.01, \end{array}$$

so that we consider the relative change in model outputs that correspond to a 1% variation in each parameter. The maximum *a posteriori* parameter vector **y**_map_ and the corresponding model outputs **o**_map_ = **G**(**y**_map_) are computed from MCMC for each of the 82 patients in the cohort, and used to compute the sensitivities in Equation (11). The resulting sensitivities are then averaged across all patients.

**Figure 5:**
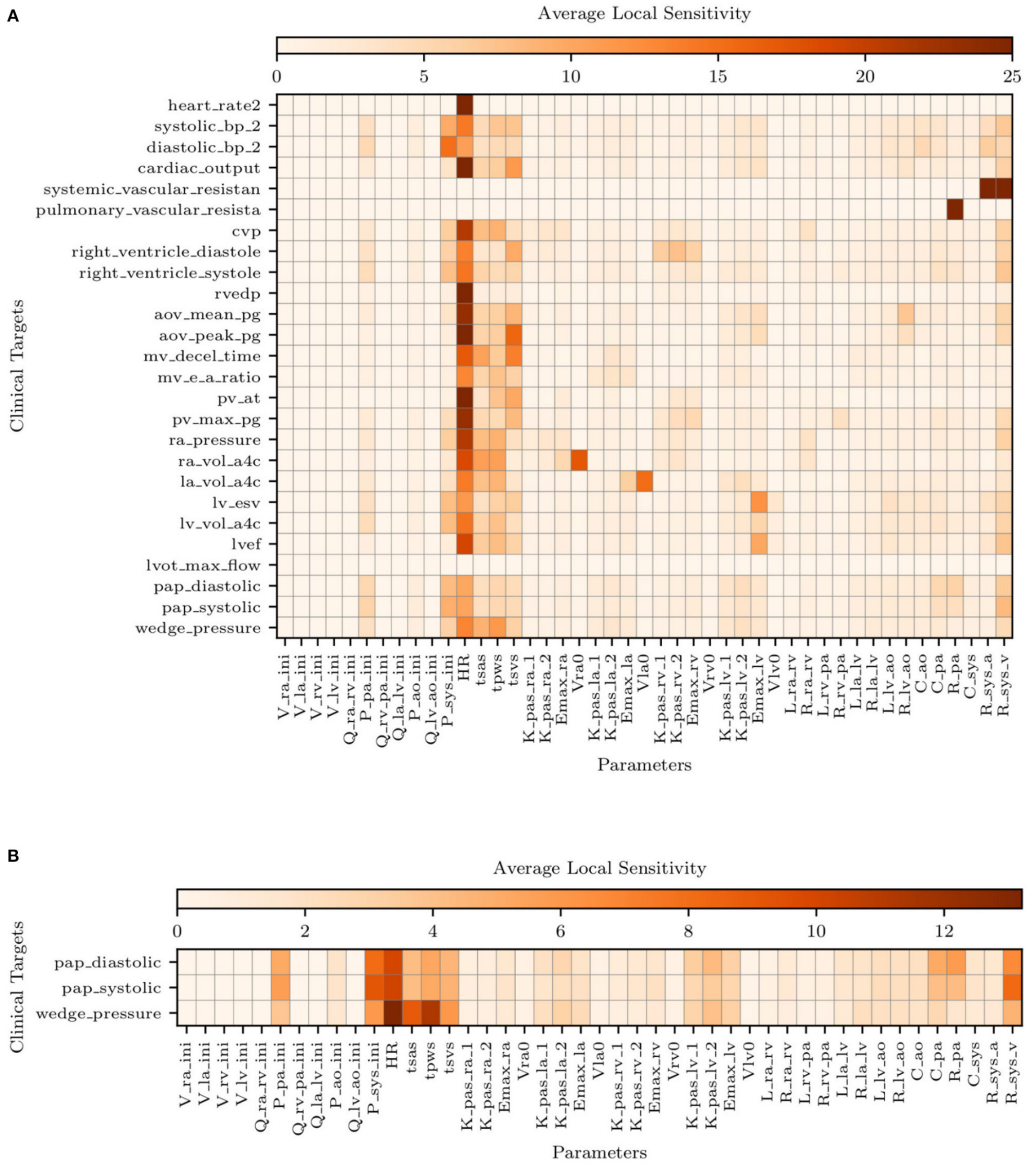
Average local sensitivities using a perturbation factor of 1%. **(A)** Average local sensitivity table for all parameters and model outputs. **(B)** Average local sensitivities table for all parameters and pulmonary outputs only. The values in each row are scaled to make their sum equal to 100.0 and two cutoffs are used equal to 25 and 12.5 for **(A,B)**, respectively.

Figure 5A illustrates the average sensitivities obtained by training our model with the complete list of clinical targets, including systolic, diastolic, and venous wedge pulmonary pressures and pulmonary vascular resistance. We note that large sensitivities are apparent for the heart rate across all outputs while at the same time, accurate measurements of heart rate are easy to obtain non-invasively. Additionally, to check how the sensitivities were affected by the availability of pulmonary pressure targets (i.e., the very same quantities we would like to predict), we re-computed sensitivities using parameter estimates **y**_map_ obtained by excluding the pulmonary pressure targets during training. The average sensitivities appear to be minimally affected by the selective exclusion of pulmonary pressure targets.

Figure 5B suggests the following important parameters. Changes in the heart rate or *t*_*svs*_ directly affect the amount of blood flow ejected by the ventricles, altering the mean pulmonary pressures under a constant PVR and initial condition (i.e., *P*_*pa,ini*_). As already discussed in the above sections, the left ventricular diastolic pressure/volume ratio *K*_*lv,pas*,1_ and the associated exponential factor *K*_*lv,pas*,2_ govern the diastolic properties of the left ventricle, while the *E*_*max,lv*_ is instead responsible for the systolic function. The left atrioventricular and aortic valve resistance *R*_*la,lv*_ and *R*_*lv,ao*_, respectively, govern the pressure drop from the left atrium to the left ventricle and from the left ventricle to the aorta. These two parameters therefore affect the left atrial and ventricular pressures and, in turn, the upstream pulmonary pressures. Mitral or aortic valve stenosis are typical examples of this mechanism (see, e.g., Tracy et al., 1990). The pulmonary resistance and capacitance parameters, *R*_*pa*_ and *C*_*pa*_, clearly affect the mean pulmonary pressures and their range. In contrast, changes in systemic vascular resistance, *R*_*sys,a*_ and *R*_*sys,v*_, affect the left ventricular afterload and, in turn the pulmonary pressures.

#### 3.3.2. Structural Identifiability

**Figure 6:**
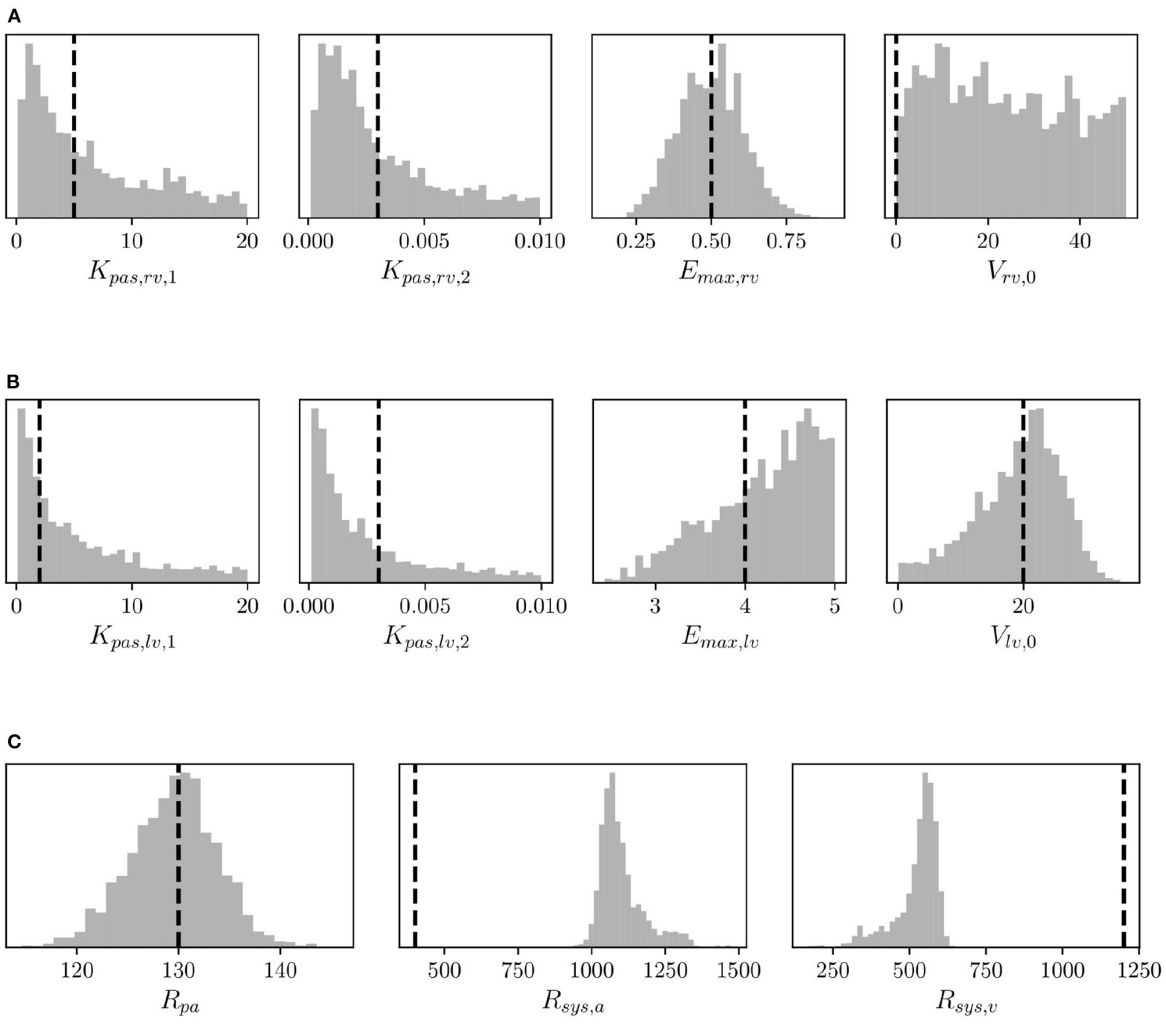
Histograms of **(A)** right ventricular, **(B)** left ventricular, and **(C)** pulmonary and systemic resistance model parameters from MCMC and default parameter values (vertical dashed lines) used to assess structural identifiability.

Structural identifiability analysis is performed to gain an understanding of our ability to recover a given set of model parameters through the solution of an inverse problem from *idealized noiseless* clinical targets that belong to the model range. This contrasts with the analysis in the next section of the *practical* identifiability, where real clinical data are used instead. We solve the model for the default parameter combination (see Supplementary Table 1) and regard the outputs as data, from which a MAP estimate of the default parameter values is re-computed by MCMC followed by NM optimization. We would like to point out that the parameters in Supplementary Table 1 correspond to a *healthy* patient. Therefore no attempt is made in this section to represent hypertensive physiological conditions. Additionally, histograms are generated using 5,000 samples from the MCMC parameter traces and compared to the default (true) parameter set. The results for right ventricular, left ventricular, and resistor parameters can be seen in Figures 6A–C, respectively. The true parameters are always found within the parameter distributions from MCMC and often correspond to the mode of the histogram. Some deviations may be observed in Figure 6C regarding the arterial and venous systemic resistance. In such a case, since SVR is the sum of such resistances, the increase in the first is compensated by a reduction in the second.

**Figure 7:**
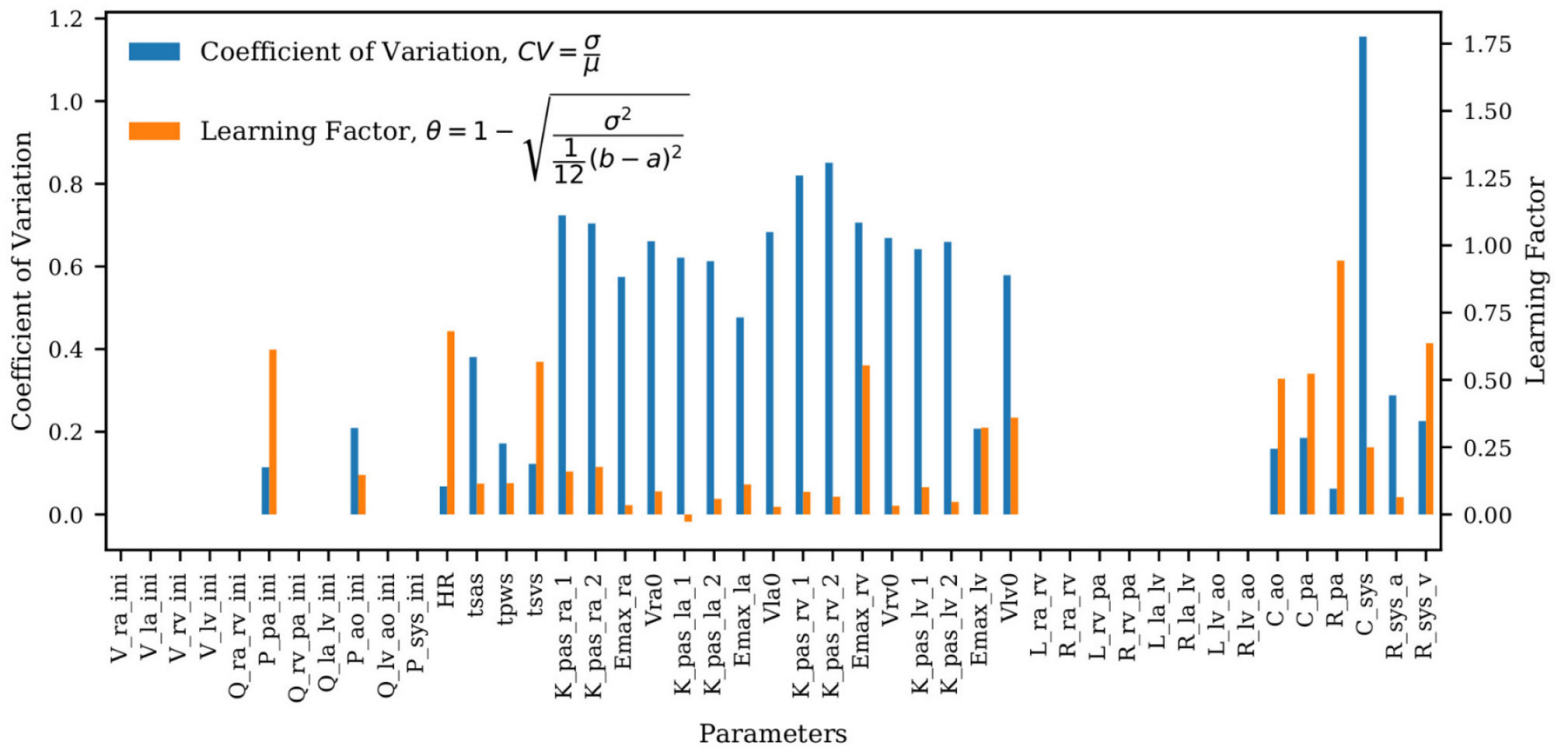
Bar plot of the coefficient of variation (*CV*) alongside the learning factor, (θ) for each parameter. *a* and *b* represent the minimum and maximum bound of each parameter uniform marginal prior.

The coefficients of variation for the model parameters marginal posteriors and associated *learning factors* are shown in Figure 7. While parameters with a higher coefficient of variation have a greater spread relative to their mean, the learning factor quantifies how much the marginal variance is reduced by conditioning the model output to the available observations or, in other words, how much the marginal variance is reduced from the prior to the posterior (Schiavazzi et al., 2016). Figure 7 shows that the parameters with the largest coefficient of variation are also the parameters with the smallest learning factor, as expected. Heart timing parameters, parameters for systemic and pulmonary resistance and compliance, the aortic compliance parameter, and the active ventricular parameters are generally well-learned.

**Figure 8:**
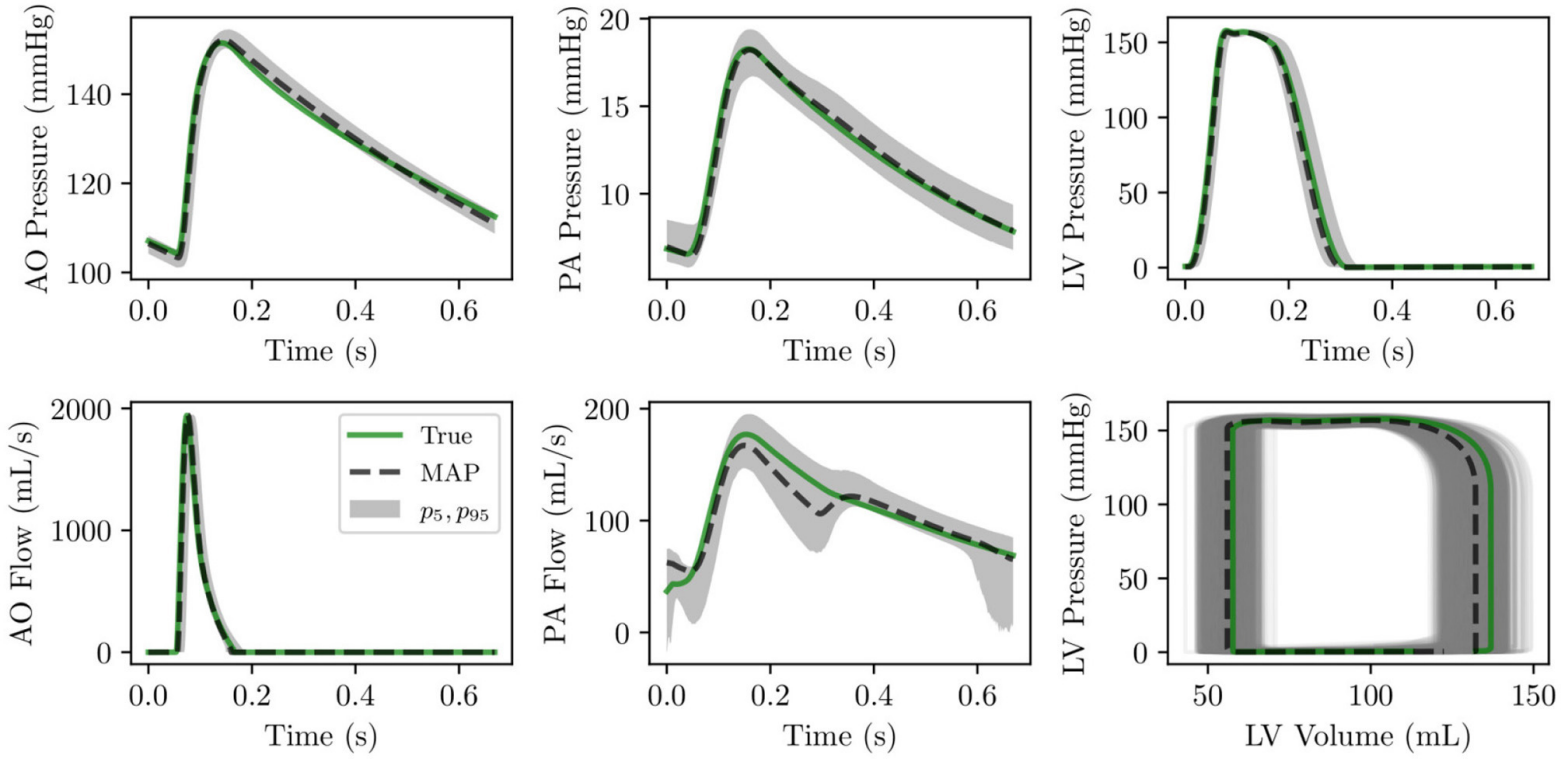
(Row 1) pressure time histories over one heart cycle for aortic, pulmonary and left ventricular pressure. (Row 2) flow time histories over one heart cycle for left ventricular outflow and pulmonary flow as well as left ventricular pressure-volume loop.

The analysis is completed by a comparison between the true and optimal physiology, calculated using 5,000 parameter combinations from the MCMC traces, and analyzed over a single heart cycle. The results for the aortic, pulmonary, and left ventricular pressures and flows, and the left and right ventricular pressure-volume loop are reported in Figure 8. Pressures, flows, and volumes agree well with those generated from the default parameter set.

#### 3.3.3. Practical Identifiability

**Figure 9:**
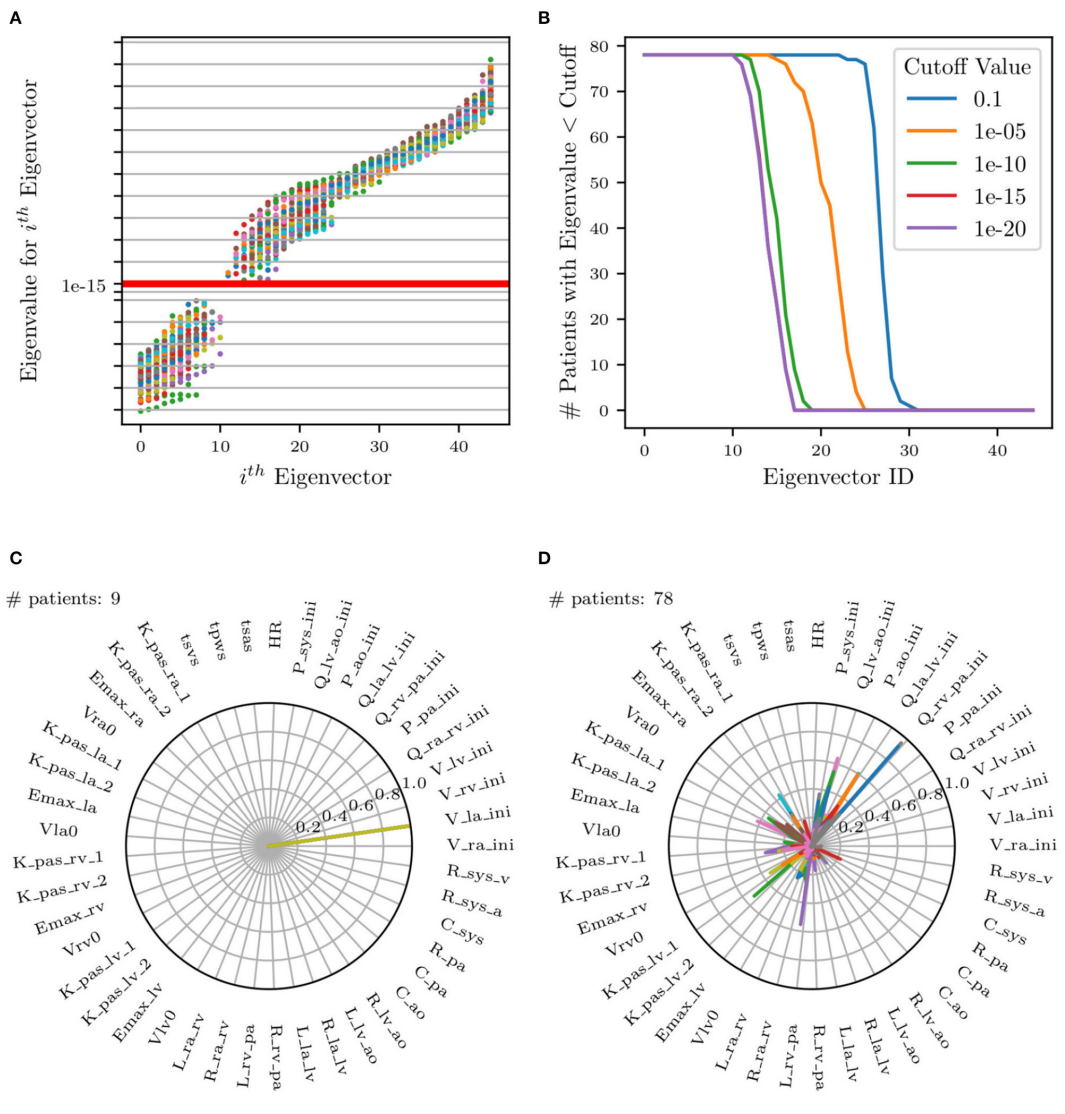
**(A)** Scatter plot of eigenvalues vs. eigenvectors for all patients. Red horizontal line represents selected cut-off value. FIM eigenvalues at identified parameters plotted in increasing order. **(B)** Plot of number of patients selected (eigenvalues less than cut-off) for each eigenvector. **(C,D)** Selection of two of the 17 total radar plots of all parameters whose eigenvalues are less than the selected cut-off. **(C)** Example of unimportant initial condition, i.e., whose perturbation has no effect on the model results. **(D)** Example of non-identifiable parameter combinations where no dominant parameter can be identified and where the combination significantly changes across patients.

We also perform local identifiability analysis through the Fisher information matrix rank (Rothenberg, 1971) to determine the presence of non-identifiable parameter combinations and unimportant parameters. To do so, we compute the matrix ∂**G**(**y**_map_)/∂**y** of local derivatives for our output quantities **o**_map_ = **G**(**y**_map_) with respect to the parameters **y**. The Fisher Information matrix $I\left({\text{y}}_{\text{map}}\right)$ can be computed as

(12) $$\begin{array}{l} I\left({\text{y}}_{\text{map}}\right)=\left[\frac{\partial \text{G}\left({\text{y}}_{\text{map}}\right)}{\partial \text{y}}\right]\text{B}{\left[\frac{\partial \text{G}\left({\text{y}}_{\text{map}}\right)}{\partial \text{y}}\right]}^{T}, \end{array}$$

where the *precision* matrix **B** contains the inverse target variances, i.e., $B_{i,i}=1/\sigma_{i}^{2}$ and *B*_*i,j*_ = 0 for *i* ≠ *j*. Rank deficiency in $I\left({\text{y}}_{\text{map}}\right)$ reveals the presence of non-identifiable parameter combinations as illustrated in Figure 9A by plotting the Fisher information matrix (FIM) eigenvalues ordered by magnitude for all patients. Small eigenvalues are observed across all analyzed patients, confirming a lack of local identifiability.

Eigenvectors for all patients whose corresponding eigenvalues were less than a selected cut-off were superimposed on the same radar plot to search for situations characterized by dominant components, representative of unimportant parameters. This process is visualized in Figure 9A, illustrating all eigenvalues colored uniquely per patient and Figure 9B, showing the effect of changing the cut-off value on the number of selected patients. Specifically, it shows that no significant changes result by adopting cut-offs in the range [1 × 10^−12^, 1 × 10^−16^].

Figures 9C,D shows an example of two of the 17 radar plots generated for this study. The plot on the left shows an example of an unimportant parameter with dominant eigenvalue associated with the parameter *V*_*la,ini*_, i.e., the initial left atrial volume. Instead, the radar plot on the right does not show any clear pattern involving parameter combinations that significantly change across patients. This local identifiability analysis confirms how initial conditions for pressures, flows, and volumes (except *P*_*pa,ini*_, *P*_*ao,ini*_, and *P*_*sys,ini*_ as per the results of the previous sensitivity analysis) are generally unimportant.

### 3.4. Prediction of Pulmonary Pressures

**Figure 10:**
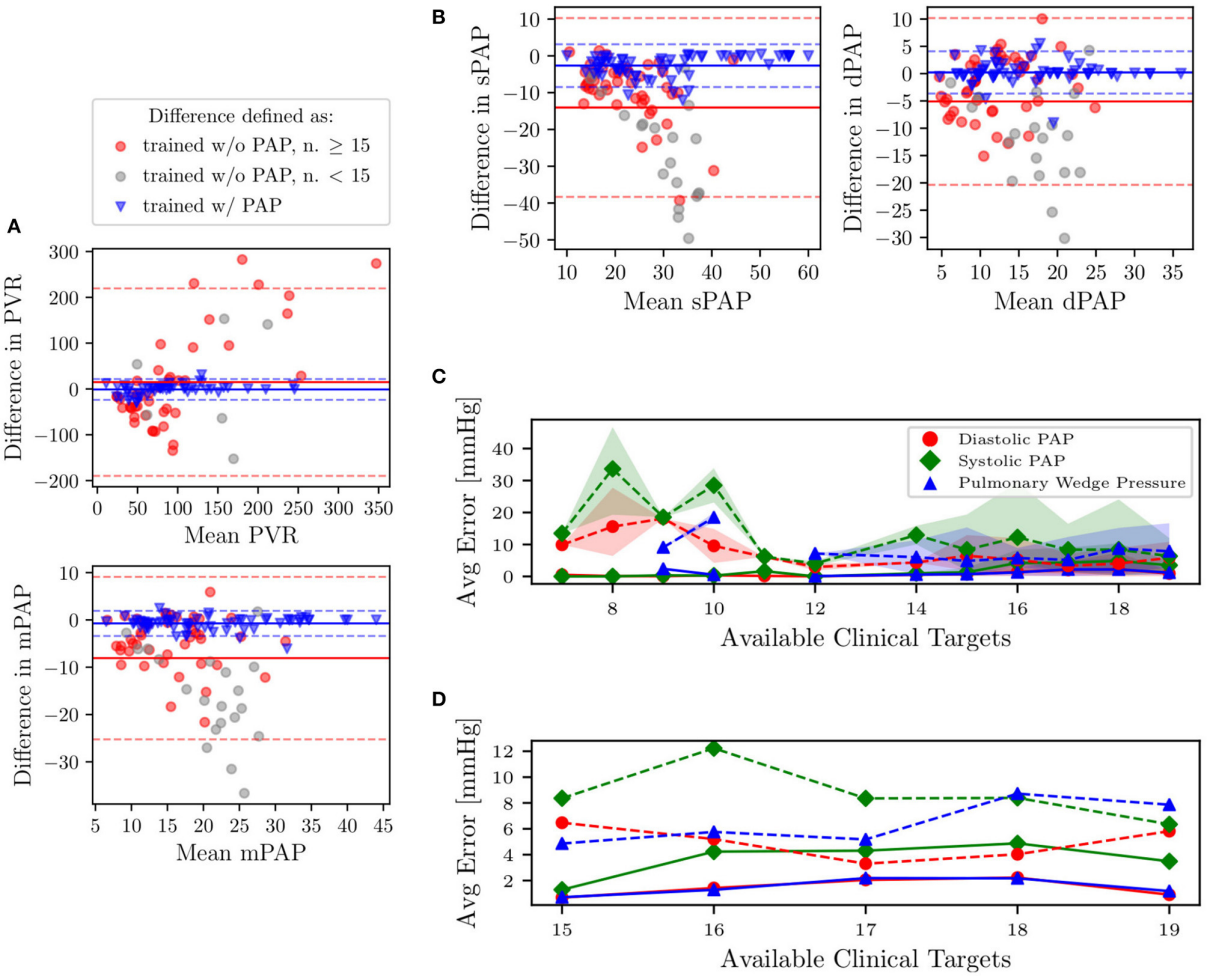
**(A,B)** Bland-Altman plots of sPAP, dPAP, mPAP, and PVR for clinical targets, and as predicted from models trained with and without pulmonary targets, respectively. Solid horizontal lines represent the mean of all differences, while dashed lines are drawn at 1.96 times the standard deviation. Predictions associated with gray markers in the Bland-Altman plots result from using less than 15 clinical targets. **(C)** Predictive performance for pulmonary pressure. Absence of PAP targets in average prediction errors is represented using dashed lines. The shaded region represents the area bounded by the 5th and 95th percentile from 5,000 random subsamples from MCMC. **(D)** Zoom on average errors which correspond to patients with more than 15 available clinical targets.

We now focus on the problem of using the physiological consistency of our compartmental model to *predict* pulmonary pressures from other possibly non-invasive clinical targets. To do so, we have trained our models without including the pulmonary targets (i.e., systolic, diastolic, wedge pressures, and pulmonary vascular resistance) and propagated forward the estimated parameters to quantify the marginal distributions of these targets. We then evaluated the error between predicted and true pulmonary pressures together with their variability. To do so, we first show four Bland-Altman plots for sPAP, dPAP, mPAP, and PVR, respectively (Figures 10A,B). Minimal bias is generated when training the model with the pulmonary pressures and PVR. Predictions generated by pulmonary pressure-blind training does not seem to generate proportional bias except for the predicted PVR.

In Figure 10C, we also plot the average absolute pressure error, with associated uncertainty, across 31 patients (those for which the pulmonary pressure was available and characterized by having more than six REDCap entries) versus the minimum number of prescribed clinical targets. Finally, as illustrated in the closeup shown in Figure 10D, which focuses on patients with at least 15 REDCap entries, the average errors on the predicted pressures is around 8 mmHg for systolic PAP, and 6 mmHg for Diastolic PAP and PCW.

### 3.5. Relative Importance of Non-pulmonary Targets

In this section, we investigate which clinical targets are the most important to include during training, in order to minimize errors in pulmonary pressure predictions. In other words, we rank clinical targets starting from those having a more beneficial impact on the accuracy in predicting pulmonary hypertension. We achieve this goal through a sequence of optimization steps. We begin by performing training using optimization with a single target at a time. The target found to minimize the average *combined prediction error* for pulmonary pressures is ranked first and permanently added to the list of targets included in all successive optimization steps. The percent error was computed for each clinical pulmonary target that was available for each patient. Percentage error expresses the percentage difference between the model output and the clinical target. A *cumulative* percentage error was calculated by taking the sum of the percentage errors for each pulmonary target clinically available for each patient. To avoid bias toward patients with fewer available pulmonary targets, the cumulative percentage error is transformed to an *average* combined prediction error through division by the number of available pulmonary targets. Let *p* ∈ {1, 2, 3, 4} be the number of pulmonary targets available in the data of a certain patient. The percent error of clinical target *s*_*i*, target_, *i* = 1, …, *p* is computed as *e*_*i*_ = 100·|*s*_*i*, target_ − *s*_*i*, output_|/*s*_*i*, target_, *i* = 1, …, *p*, while the average combined prediction error is $\overset{p}{\underset{i=1}{\sum }},e_{i}/p$.

**Figure 11:**
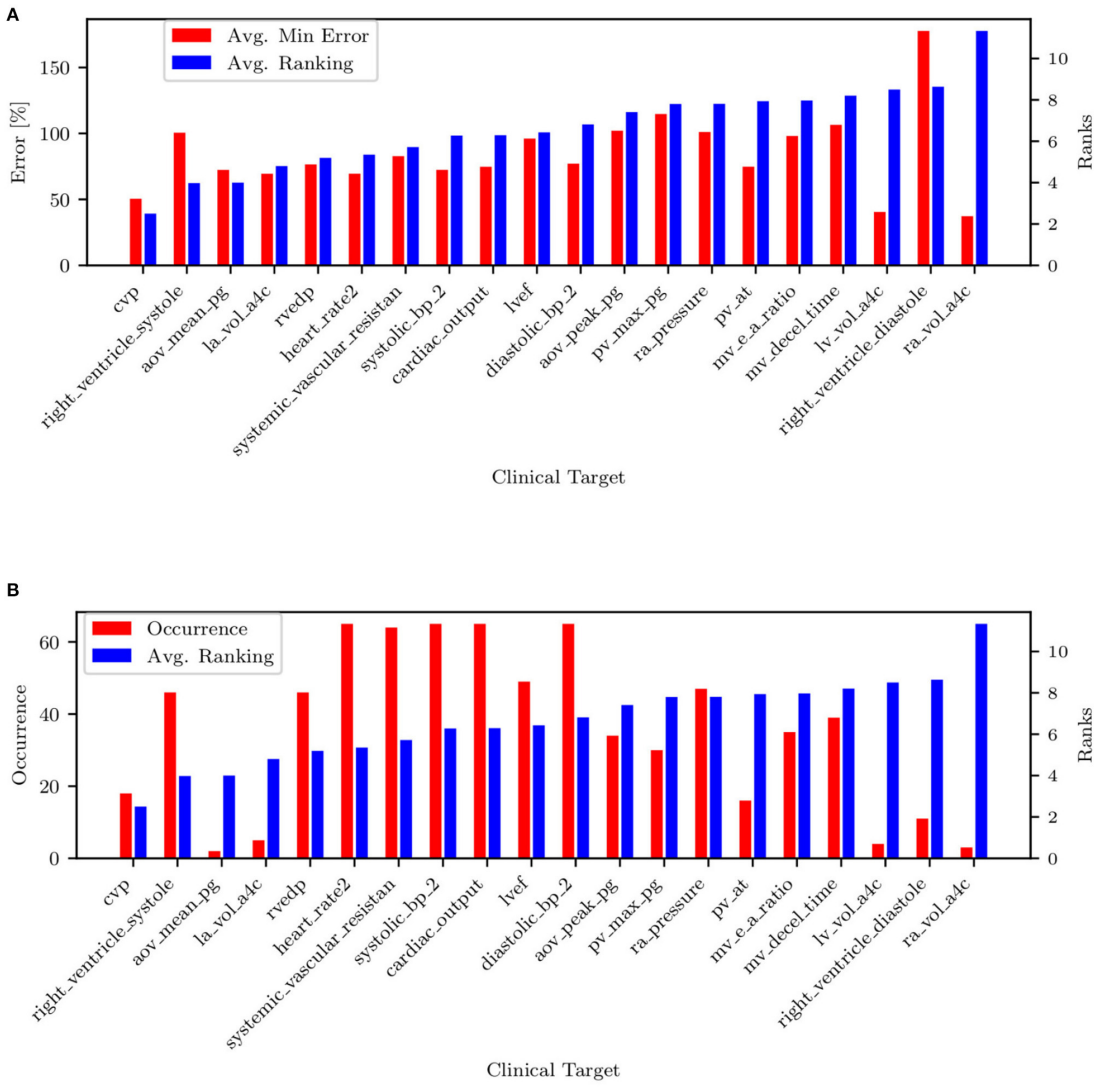
**(A)** Target ranking with minimum prediction error. **(B)** Target ranking and associated occurrence.

All remaining targets are iteratively tested, and those producing the minimum average combined PAP errors (shown in Figure 11A) are progressively ranked until all targets have been considered. The process above is repeated for each patient. Figure 11B shows the resulting average ranking and associated occurrences, i.e., the number of patients where a specific target was collected.

**Figure 12:**
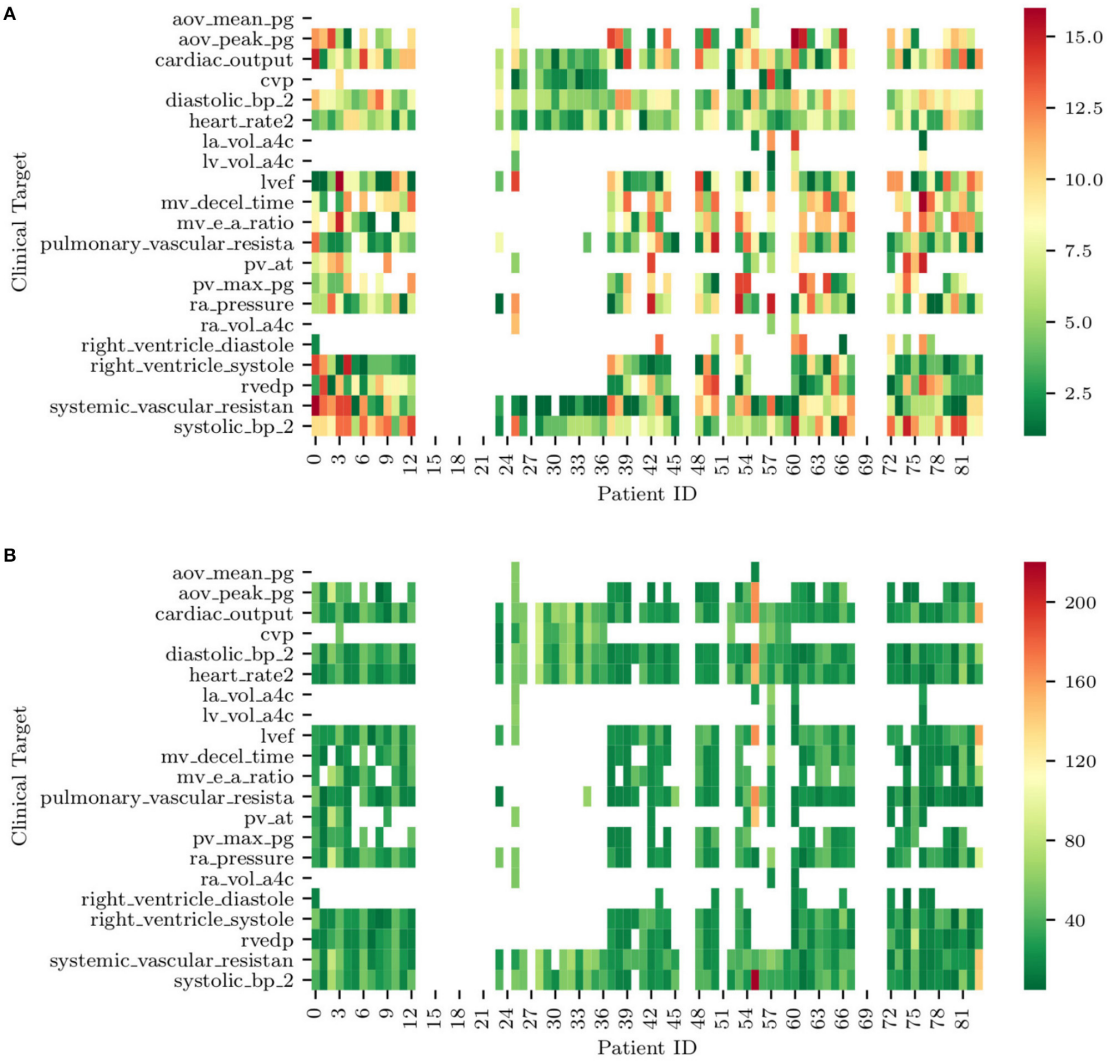
**(A)** Ranks of clinical targets for each patient in order of importance (i.e., green more important, red less important). **(B)** Minimum combined prediction error associated with the introduction of each clinical target for all the patients included in the study. Note how blank entries correspond to missing clinical targets.

The list of targets ordered by average rank and filtered by occurrence is also shown in Table 5. Most of the quantities (except mPAP, PCW, PVR, and SVR) can be estimated non-invasively. From RAP (or CVP, RVEDP), SBP-DBP, HR, and SVR it is possible to estimate the cardiac output. From CO, HR, and LVEF, it is possible to estimate LVEDV, which is correlated with PCW. This might explain why PCW is always estimated better than sPAP and dPAP. The mitral valve deceleration time and velocity E/A ratio are indicators of diastolic LV function and therefore correlated with PAP. If PVR, CO, and PCW are known, it is possible to estimate mPAP, which is correlated to sPAP and dPAP. Finally, the parameter ranking is summarized for all patients in Figure 12.

**Table 5:** Target list in order of average rank, filtered by occurrence.

**Ranks**	**Clinical target**	**Description**
1	cvp	Central venous pressure
2	right_ventricle_systole	Right ventricle systolic pressure
3	rvedp	Right ventricle end diastolic pressure
4	heart_rate2	Heart rate
5	systemic_vascular_resistan	Systemic vascular resistance
6	systolic_bp_2	Systolic brachial pressure
7	cardiac_output	Cardiac output
8	lvef	Left ventricular ejection fraction
9	diastolic_bp_2	Diastolic brachial pressure
10	aov_peak_pg	Peak pressure gradient across aortic valve
11	pv_max_pg	Peak pressure gradient across pulmonary valve
12	ra_pressure	Mean right atrial pressure
13	pv_at	Pulmonary valve acceleration time
14	mv_e_a_ratio	Mitral valve E/A ratio
15	mv_decel_time	Mitral valve deceleration time

*Targets with <5 occurrences were excluded from the ranking order*.

### 3.6. PH Classifiers From Assimilated Circulation Models

This section explores the use of trained lumped parameter models for the automatic detection of abnormal pulmonary pressures from minimally invasive clinical measurements. We employ a naïve Bayes classifiers (see, e.g., Lewis, 1998) to detect pulmonary hypertension from our dataset. The first step of this classification process was to generate a ground truth variable for hypertension that could be used to analyze the accuracy of our hypertensive classification. Using the patient's clinical data, a binary hypertensive variable was defined using the criteria from Simonneau et al. (2013). If a patient had mPAP > 25 mmHG or sPAP > 35 mmHG, then the patient was classified as hypertensive. Of the 82 patients, 65 of the patients contained sufficient clinical data to define a ground truth binary hypertensive variable. The remaining 17 patients did not contain enough clinical data to determine a ground truth value for hypertension, so these patients were excluded from the testing and training datasets.

**Figure 13:**
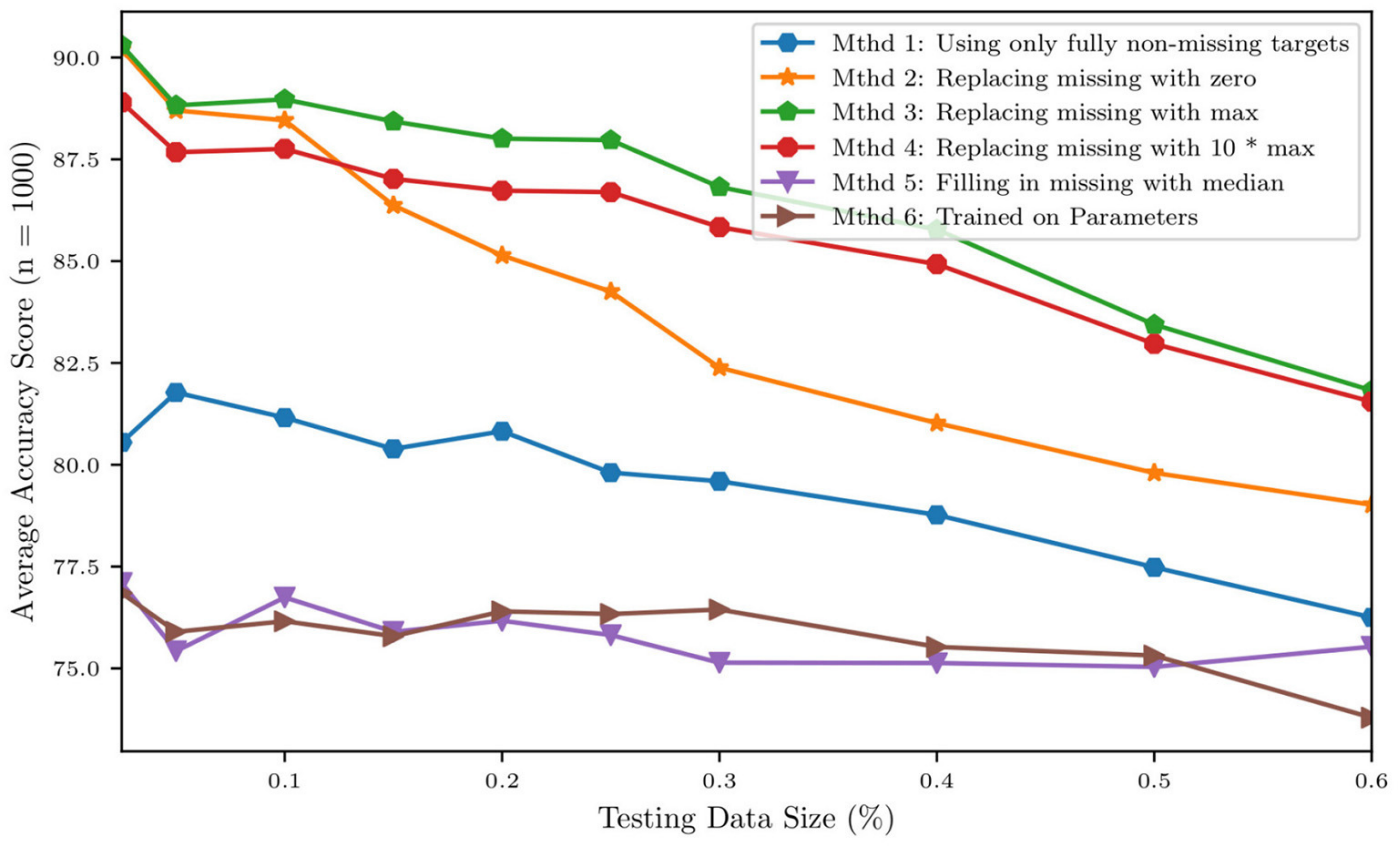
Accuracy of a naïve Bayes classifiers for pulmonary hypertension using different approaches for multiple imputation.

Before a naïve Bayes classification can be performed, the problem of missing data must first be addressed. Our dataset representing the cohort of 82 patients contains a non-negligible ratio of missing data, with patients missing between 1 and 19 of the 24 total clinical targets. To overcome this issue and to verify how classification results depend on the strategy selected for missing data imputation, five different missing data imputation approaches were tested. The first method tested was *complete-case analysis*, considering only the 4 variables that were available for all patients, i.e., heart rate, systolic blood pressure, diastolic blood pressure, and cardiac output. The remaining four missing data methods consisted of replacing the missing values with (1) zeros, (2) the max value of the data set, (3) a value far outside the range of the data set (10 times the max), or (4) the median. In addition to the training of five separate classifiers using five different missing data methods, a sixth naïve Bayes classifier was trained on the MAP parameters of the model. The data for the MAP parameters from the model is a complete set, so no missing data method was required. Figure 13 shows how the above imputation approaches affect classification accuracy. At first sight, the high accuracy produced by a multiple imputation strategy using the maximum inter-patient clinical value (or 10 times its value) may seem surprising. This can be explained by observing how, in such a case, the probability of the feature associated with the missing data is practically zero, thus essentially reducing the number of features and increasing the resulting accuracy.

We then grouped patients according to a training and a testing set, consisting of 4/5 and 1/5 of the available data, respectively. Training and testing is performed either using the raw clinical data or model parameters assimilated though optimization and MCMC, resulting in ~88% accuracy for the classifier trained with the 24 raw clinical data points and about 76.4% accuracy for the classifier trained with the 45 assimilated parameter values (see Table 6).

**Table 6:** Training accuracy improvements using the original dataset and following pre-processing to balance the relatively small number of hypertensive positives.

		**Pre-processing data for classification**		
		**Complete data**	**Missing data imputed**	**Balanced data**
Training data type for classifier	Clinical data	0.8082	0.8801	1.0
	Parameters	0.7640	N.A.	1.0

Of the 82 patients, 65 contained enough information for ground truth classification of whether the patient should be identified as hypertensive. Based on these data, we were able to classify 22 of the 65 patients, or about one-third of the patients, as being affected by pulmonary hypertension and the remaining two-thirds with normal PAP pressures. This imbalance is known to introduce bias (Sun et al., 2009), with the classifier more likely to label patients as non-hypertensive (as 2/3 of the training data are non-hypertensive). As a remedy, six different approaches were applied to deal with this unbalance, which represents a mixture of over-sampling methods, under-sampling methods, and a combination of both.

**Figure 14:**
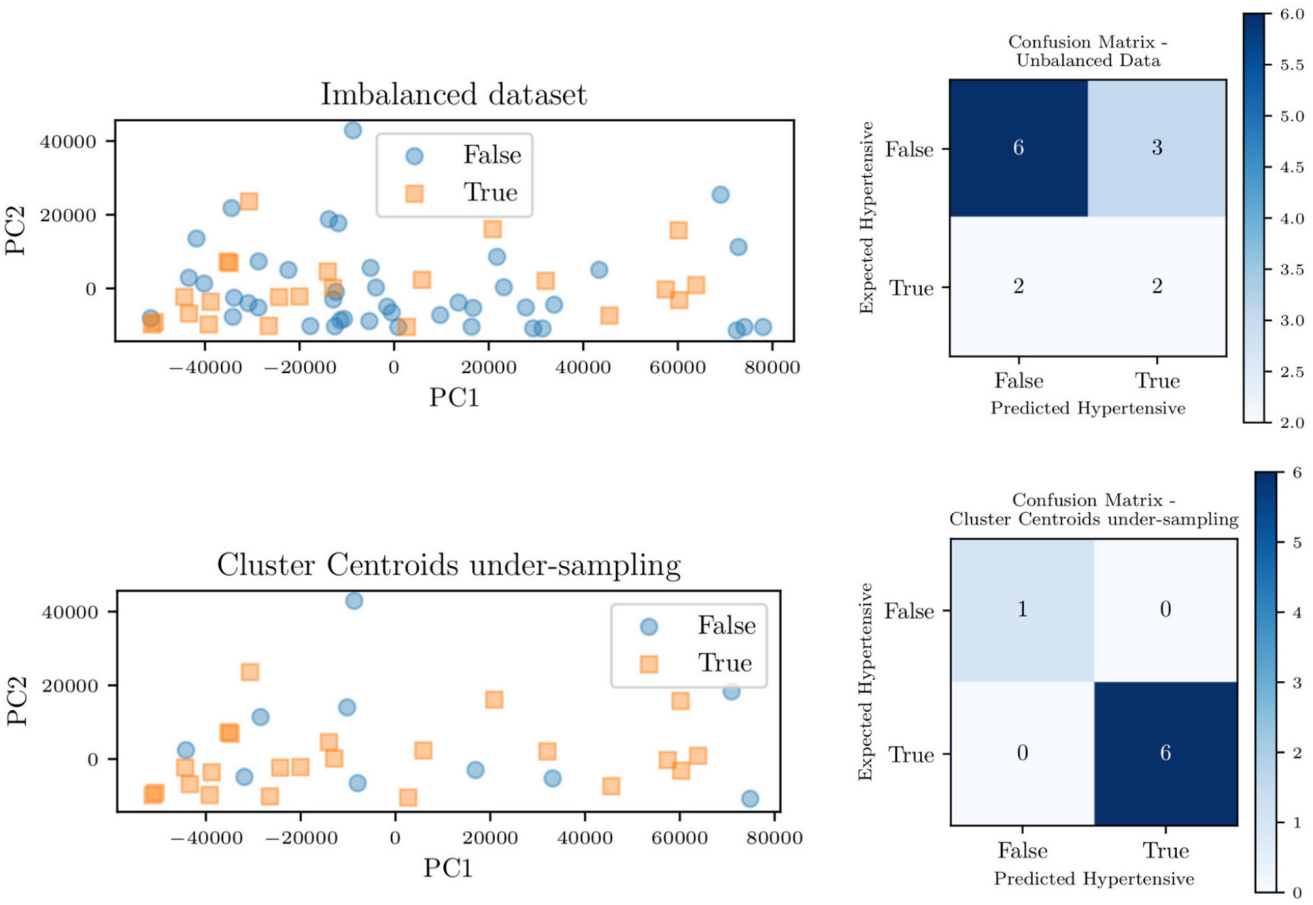
Principal component decomposition (left) along with the contingency table (right) for the unbalanced data and balanced through *centroid clustering*.

Figure 14 shows the principal component decomposition and contingency table for the unbalanced data and the data balanced with centroid clustering. Note how the confusion matrices are evaluated based on test data, hence 13 patients (1/5 of the 65 patients) for the unbalanced dataset. Undersampling in centroid clustering reduces the training/testing data from 65 to 33: hence one-fifth of the 33 samples becomes ~7, as shown in the confusion matrix for the centroid clustering under-sampling method.

**Figure 15:**
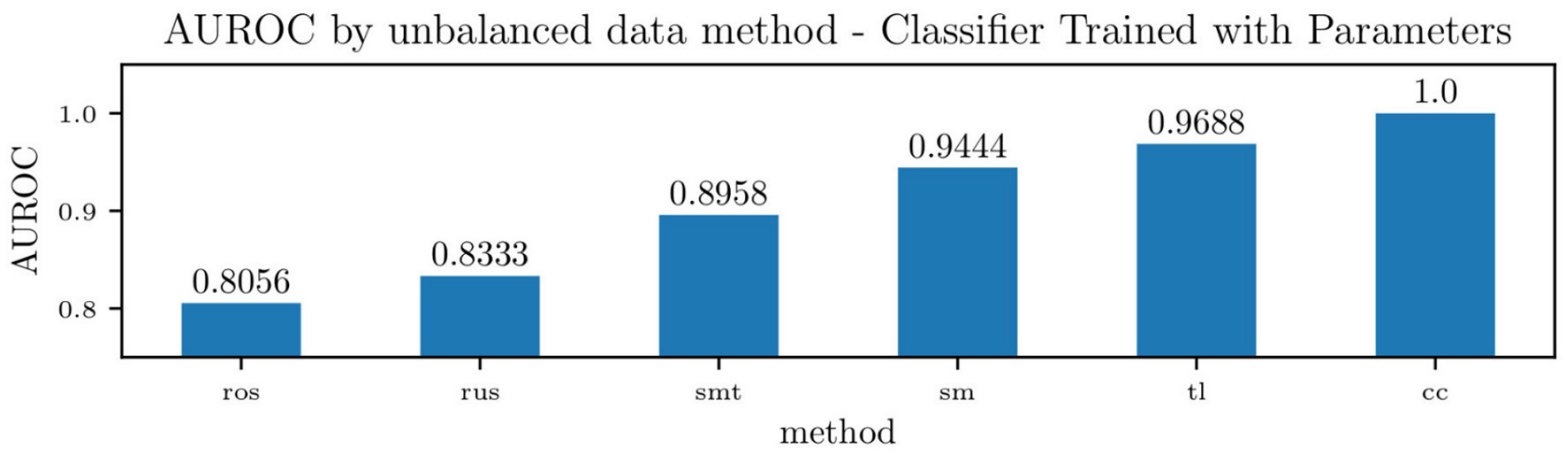
Accuracy of a Naïve Bayes classifiers for pulmonary hypertension, using identified lumped parameters as features, and various methods to handle unbalanced data. The methods used were Random Over-sampling (ros), Random Under-sampling (rus), an over-sampling method: SMOTE (Synthetic Minority Oversampling TEchnique) (sm), two under-sampling methods: Tomek Links (tl) and Cluster Centroids (cc), and a hybrid approach that performs over-sampling followed by under-sampling using SMOTE followed by Tomek Links (smt).

Figure 15 shows the effect of each unbalanced data method on the overall area under the receiver operating characteristic curve (ROC) for the classifier trained on the model parameters. For such a classifier, centroid clustering (Lin et al., 2017) increases accuracy from 76.4% up to 100%. In addition, data balance using Synthetic Minority Oversampling TEchnique (SMOTE) (Chawla et al., 2002) increases the accuracy of the model trained on the data from 88 to 100%, as shown in Table 6. We again remark that the number of patients in this study is small, and future studies will investigate the generalization of this approach to larger datasets.

## 4. Conclusion

This study demonstrates that a relatively simple lumped parameter compartmental model can represent a wide range of physiologies, spanning healthy patients to patients affected by severe diastolic left ventricular dysfunction. In this context, an activation formulation for the heart chambers has proven important to separately account for systolic and diastolic pressure-volume behavior. When trained using ideal clinical data from subjects with diastolic left ventricular dysfunction, the parameters associated with the governing physiological mechanisms (i.e., diastolic ventricle relaxation) change in a way that is consistent with the underlying physiology of the dysfunction. The average parameter sensitivities are determined after training with real data from 82 patients, confirming intuition for the most important parameters. Pulmonary pressures were found to be primarily sensitive to the following parameters: heart rate and contraction timing parameters, diastolic and systolic pressure/volume ratio parameters (*K*_*pas*, 1, *lv*_, *K*_*pas*, 2, *lv*_, and *E*_*max,lv*_), left atrioventricular and aortic valve resistance (*R*_*la,lv*_, *R*_*lv,ao*_), pulmonary resistance and capacitance (*R*_*pa*_, *C*_*pa*_) and systemic resistance (*R*_*sys*_). Additionally, the model was found to be locally unidentifiable, with initial conditions generally unimportant. The structural identifiability analysis showed that inference of model parameters is feasible under perfect, noiseless conditions and with a sufficiently large number of available clinical targets. Target ranking based on sequential optimization reveals the most important non-invasively acquired clinical targets: heart rate, systemic pressures, peak pressure gradient across aortic and pulmonary valves, pulmonary valve acceleration time, mitral valve deceleration time, and mitral valve E/A peak ratio. The clinical targets that positively affect the prediction of pulmonary pressures, but require an invasive practice for their measurement, are central venous pressure, right ventricular systolic and diastolic pressure, systemic vascular resistance, cardiac output, and mean right atrial pressure. After investigating parameter identifiability/sensitivity, the pulmonary pressures of the 82 patients with various heart failure severities were predicted from a lumped hemodynamic model, which was trained based on the remaining clinical targets. The average absolute pressure error on the 11 patients characterized by at least 11 distinct clinical entries was found ≃8 and 6 mmHg for systolic and diastolic/wedge pulmonary pressures, respectively. While these errors may seem large to detect cases of mild hypertension, they may be more than reasonable depending on how high are the PA pressures we are trying to predict/detect. In other words, while an 8 mmHg pressure error may seem relevant with an actual pulmonary pressure of 20 mmHg, for cases of patients with the *real* disease (i.e., PA pressures of 40, 50, 60 s, etc.), then the difference between (say) 50 and 58 would not be nearly as large. However, more importantly, our approach would still identify those patients as patients that would require further evaluation.

Finally, we have shown that MAP estimates of circulation model parameters can be used to detect elevated pulmonary pressures, and that a simple classifier provides high accuracy on balanced data, even when these parameters are identified without measuring systolic, diastolic, wedge pulmonary pressures, and pulmonary vascular resistance. Future work will be devoted to the systematic demonstration of the proposed approach on larger patient data sets, opening new avenues for translational applications of model-based diagnostics, improving model formulations that target specific diseases, and developing improved and more efficient estimation approaches.

## Data Availability Statement

The datasets analyzed for this study plus a Python/Cython implementation of the adult circulation model are available at https://github.com/desResLab/supplMatHarrod20.

## Ethics Statement

The study was classified as research not involving human subjects and approved on June 13th, 2019 by the Office of Research Compliance and Institutional Review Board at the University of Notre Dame under IRB#19-05-5371. All procedures performed in studies involving human participants were in accordance with the ethical standards of the institutional and/or national research committee and with the 1964 Helsinki declaration and its later amendments or comparable ethical standards. Written informed consent for participation was not required for this study in accordance with the national legislation and the institutional requirements.

## Author Contributions

DS, AM, and KH designed the numerical experiments. JR provided the dataset and initial supervision on the study. DS implemented the physiological model and developed the tulip library. KH performed the computer simulations and prepared the images and tables. JF provided clinical supervision. All authors reviewed the article before publication.

## Conflict of Interest

The authors declare that the research was conducted in the absence of any commercial or financial relationships that could be construed as a potential conflict of interest.
