# Supplementary material for: Predictive Modeling of Secondary Pulmonary Hypertension in Left Ventricular Diastolic Dysfunction
Source: Front Physiol. 2021 Jul 1;12:666915. doi: 10.3389/fphys.2021.666915 (PMC8281259; doi:10.3389/fphys.2021.666915)
Supplement: Supplementary file 1 [file Data_Sheet_1.pdf]

Supplementary Material

1 UNIFORM PRIORS AND DEFAULT PARAMETER SET

Table S1 shows the maximum and minimum ranges for each lumped parameter. The default parameter values used for the structural identifiability analysis are also reported in column *Default*.

Table S1. Maximum and minimum values defining the priors for the model parameters.

Parameter	Symbol	Default$^{(*)}$	Min$^{(*)}$	Max$^{(*)}$
Heart Rate	HR	78.0	40.0	100.0
Atrial relative activation duration	t_{sas}	0.2	0.05	0.4
Atrial relative activation time shift	t_{pws}	9.5	5.0	10.0
Ventricular relative activation duration	t_{svs}	0.4	0.1	0.5
Atrial passive curve slope, right atrium	$K_{pas,ra,1}$	5.0	0.1	10.0
Atrial passive curve exponent factor, right atrium	$K_{pas,ra,2}$	0.006	0.0001	0.06
Atrial active curve slope, right atrium	$E_{max,ra}$	0.1	0.05	5.0
Unstressed right atrial volume	$V_{ra,0}$	0.0	0.0	50.0
Atrial passive curve slope, left atrium	$K_{pas,la,1}$	5.0	0.1	10.0
Atrial passive curve exponent factor, left atrium	$K_{pas,la,2}$	0.0065	0.0001	0.06
Atrial active curve slope, left atrium	$E_{max,la}$	0.2	0.05	5.0
Unstressed left atrial volume	$V_{la,0}$	0.0	0.0	50.0
Ventricular passive curve slope, right ventricle	$K_{pas,rv,1}$	5.0	0.1	20.0
Ventricular passive curve exponent factor, right ventricle	$K_{pas,rv,2}$	0.03	0.0001	0.01
Ventricular active curve slope, right ventricle	$E_{max,rv}$	0.5	0.1	5.0
Unstressed right ventricular volume	$V_{rv,0}$	0.0	0.0	50.0
Ventricular passive curve slope, left ventricle	$K_{pas,lv,1}$	2.0	0.1	20.0
Ventricular passive curve exponent factor, left ventricle	$K_{pas,lv,2}$	0.003	0.0001	0.01
Ventricular active curve slope, left ventricle	$E_{max,lv}$	4.0	1.0	5.0
Unstressed left ventricular volume	$V_{lv,0}$	20.0	0.0	50.0
Inductance of right atrium	$L_{ra,rv}$	0.1	0.1	0.1
Resistance of right atrium	$R_{ra,rv}$	10.0	10.0	10.0
Inductance of right ventricle	$L_{rv,pa}$	0.1	0.1	0.1
Resistance of right ventricle	$R_{rv,pa}$	15.0	15.0	15.0
Inductance of left atrium	$L_{la,lv}$	0.1	0.1	0.1
Resistance of left atrium	$R_{la,lv}$	8.0	8.0	8.0
Inductance of left ventricle	$L_{lv,ao}$	0.1	0.1	0.1
Resistance of left ventricle	$R_{lv,ao}$	25.0	25.0	25.0
Aortic capacitance	C_{ao}	1.0e-3	1.0e-5	0.001
Pulmonary capacitance	C_{pa}	4.0e-3	100.0e-6	0.01
Pulmonary resistance	R_{pa}	130.0	1.0	500.0
Systemic capacitance	C_{sys}	400.0e-6	100.0e-6	0.05
Systemic Resistance - Arteries	$R_{sys,a}$	400.0	100.0	800.0
Systemic Resistance - Veins	$R_{sys,v}$	1200.0	500.0	2500.0

(*)All units are in the CGS system.

2 MCMC CONVERGENCE FOR STRUCTURAL IDENTIFIABILITY ANALYSIS

This section reports the convergence of the selected adaptive MCMC sampler for structural identifiability. Figure S1 shows the Gelman-Rubin MCMC convergence metric for all simulation parameters.

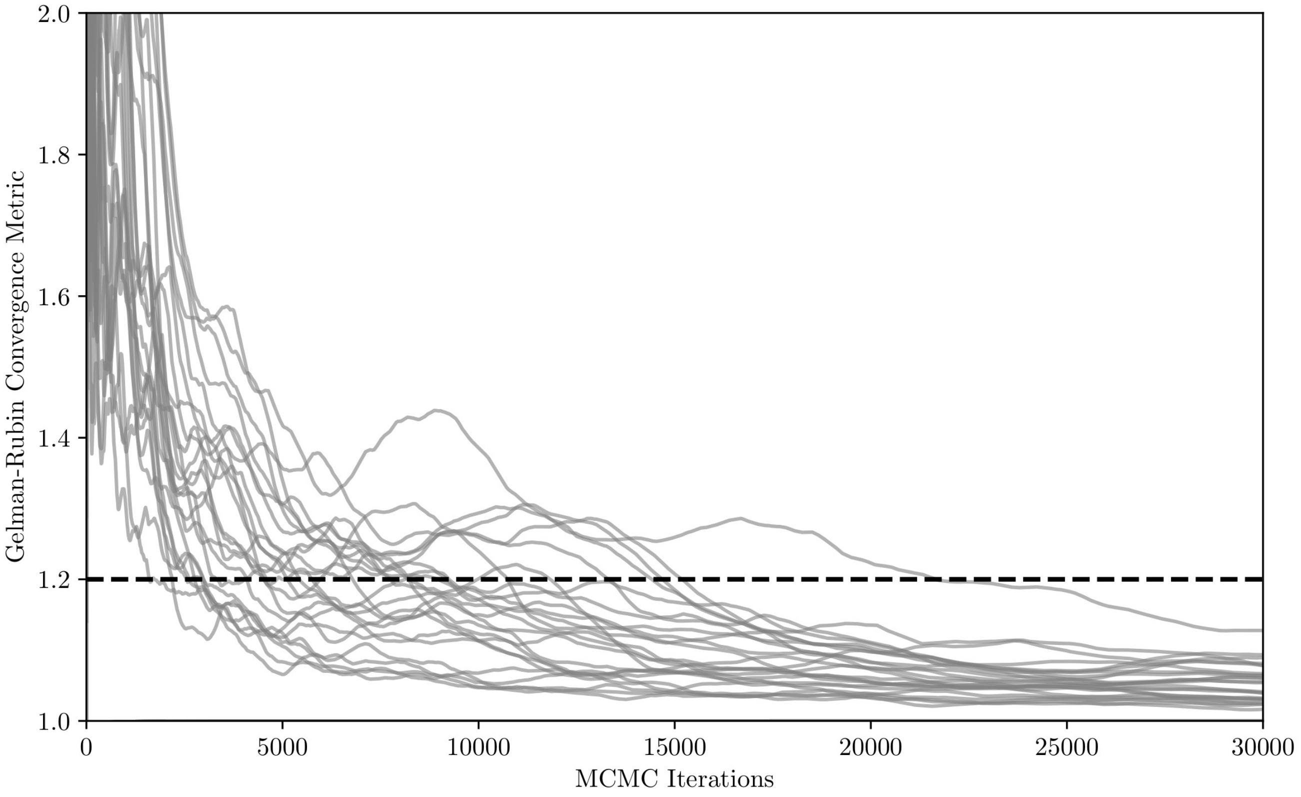

Figure S1. Plot of the Gelman-Rubin MCMC convergence metric for all simulation parameters from structural identifiabilty analysis.
